# Supplementary material for: The whole-genome and expression profile analysis of WRKY and RGAs in Dactylis glomerata showed that DG6C02319.1 and DgWRKYs may cooperate in the immunity against rust
Source: PeerJ. 2021 Aug 19;9:e11919. doi: 10.7717/peerj.11919 (PMC8380429; doi:10.7717/peerj.11919)
Supplement: Supplemental Information 1 [file peerj-09-11919-s001.docx]

**Table S1:** The sequences for BLASTP.

| gene id | sequence |
| --- | --- |
| APT42877.1 WRKY [Triticum aestivum] | MSMAPYEKVMDDLAKGQQFATQLQGLLRDSPKAGHIMDQILHTFSRAIHAAKAAAAASASESEVTDGASSGGKRKSAAGGGPRKACRTRTQDSSVVTKNMKSLEDGQTWRKYGQKEIQNSKHSKAYFRCTHKYDQQCMARRQAQRCDDDPDTFRVTYIGVHTCRDPAAAVAPHAPHLTGTAAGCHLISFAPAAAHGTTTTTNTNLVDDAAATGSGLQLPGLKLEGGDQEEVLSSRTPGSSALYGAAAAAAWPDQGDVTSTLQYGGAGAFGGGLFDGYPYLEDLLSYDLDH |
| AGX26172.1 WRKY [Oryza sativa Indica Group] | MDGARQESREYWRDGGDVVGEELLREILEETAAVHSNSNSNSNSNSNSKEAEEEDEREYFAAAAADEQLQVEAPCGRRRRESMVNKLISTVYSGPTISDIESALSFTAAGDHQLLADGHNFAASSCSPVVFSPEKTLSKTMENKYTLKIVSCGNDGGLADDGYKWRKYGQRSIKNSPNPRSYYRCTNPRCNSKKQVERAVDEPDTLIVTYEGLHLHYTYSHFLHSTSSSSSSTTTQQQLQPQPQMMTNCKKKPKLHLHPLLHDDPRPPPPPPEMTTMMIMQSFSIQQQQHDDDQLLQPAADDHLMVQAPPDDCYNINGSSSSGLMMSLEDDEQAAGAGGLLEDVVRLLVRRPPPPICNNNNYYYSPATTCTSDNEYGSSASASPSSSVSVSSWTTPMSPCIDMAILSNIF |
| NP_001030634.1 WRKY DNA-binding protein 39 [Arabidopsis thaliana] | MEEVEAANRSAIESCHGVLNLLSQRTSDPKSLTVETGEVVSKFKRVASLLTRGLGHGKFRSTNKFRSSFPQHIFLESPICCGNDLSGDYTQVLAPEPLQMVPASAVYNEMEPKHQLGHPSLMLSHKMCVDKSFLELKPPPFRAPYQLIHNHQQIAYSRSNSGVNLKFDGSGSSCYTPSVSNGSRSFVSSLSMDASVTDYDRNSFHLTGLSRGSDQQHTRKMCSGSLKCGSRSKCHCSKKRKLRVKRSIKVPAISNKIADIPPDEYSWRKYGQKPIKGSPHPRYIYKHLLV |
