# Supplementary material for: The whole-genome and expression profile analysis of WRKY and RGAs in Dactylis glomerata showed that DG6C02319.1 and DgWRKYs may cooperate in the immunity against rust
Source: PeerJ. 2021 Aug 19;9:e11919. doi: 10.7717/peerj.11919 (PMC8380429; doi:10.7717/peerj.11919)
Supplement: Supplemental Information 2 [file peerj-09-11919-s002.docx]

**Table S2:** Protein sequences of *DgWRKYs*.

| Gene id | Protein sequences |
| --- | --- |
| >DgWRKY83.1 | MAASLGLNPESLFSSAYAYSSPFLADHYAPPAGDGGFSVQSYNLHDDHQQHYRFEHSPPTAPPPISFTSAGEDERRSSEETTKATVGAGRIGFRTKSEAVEILDDGFKWRKYGKKAVKNSANPRNYYRCSAEGCGVKKRVQRDQDDPRYVVTTYDGVHNHATPGAAVAEYYCYSPARGSSGSPPAAYSAAGPLYF |
| >DgWRKY28.1 | MDNGEGSSPTDSGGLLPLFARSLQAEGLEEKVRRVSEENRRLAAMLGAILADHPHLRSLATSPASAIAATAARTTRSCSAANVAREEIIAVNVEPQPKVRTVCARAEPSGSDANLIVKDGYQWRKYGQKVTRDNPYPRGYFRCAFAPSCPVKKKVQRDADDTSLLVATYEGEHNHAKSPIGESVGNMSTGKAGSLPCSISHNFLGQMITLDLTNQDSPVTVQAASREVVTPEFQKLLVNEMVNSLKNDMEFMHAVMSAVSEKILESIPNCSS |
| >DgWRKY27.1 | MDTARRSPVCLDLMVGLPMVREPSPARGTGMGNGADIASPACGRAASMTNDEAKVMEAKLTQMSEENRRLTEMIAFLYGSQMARQSLEGEAGEPPASTAASPTSRKRSRESTDTSNSGDADSTKKTGTVEADHVDVQSPVSESTCRRIKVRKVCQPIDPSDTSLVVKDGYQWRKYGQKVTRDNPSPRAYFRCAFAPSCPVKKKVQRSAEDSSVLEATYEGEHNHPHPSRSGELIPTCATPSGAVPCSISMNSSGPTITLDLTKNGGGVVEAQPDLKKACMEATSSPEFQRALVEEMARELTADQKFTEALAAAILRKLPDY |
| >DgWRKY26.1 | MDTARRSPVCLDLMVGLPMVREPSPARGTGMGNGADIASPACGRAASMTNDEAKVMEAKLTQMSEENRRLTEMIAFLYGSQMARQSLEGEAGEPPASTAASPTSRKRSRESTDTSNSGDADSTKKTGTVEADHVDVQSPVSESTCRRIKVRKVCQPIDPSDTSLVVKDGYQWRKYGQKVTRDNPSPRAYFRCAFAPSCPVKKKVQRSAEDSSVLEATYEGEHNHPHPSRSGELIPTCATPSGAVPCSISMNSSGPTITLDLTKNGGGVVEAQPDLKKACMEATSSPEFQRALVEEMARELTADQKFTEALAAAILRKLPDY |
| >DgWRKY48.1 | MSSYSSFLSMCPVDQIGGYVDDGYNDDDMVVAATYLSSFDFDVGEQYASLPEAATAAAFPAEQQAPAPAPLLGHSPQADADSYSSGKAASSSSAGLSYQESISKCLTSGGARSKGSKIAFKTRSEVEVLDDGYRWRKYGKKMVKNSPNPRNYYRCSSEECRVKKRVERDRDDARFVITTYDGVHNHPAPLPPRGCARYALAQMHMRVEDVDTMRAHAVGQEGSTPVPAPRTVQRDD |
| >DgWRKY81.1 | MYALVPQLGGPSSVLDCRKRKGVAAEHDLGEMDEVQSQITEAFRLAGELMDELPTARDDPAYLAARCHGIVHAYSLAIRMLQGYGVGGMDVAAAAPQQFSGEPLDLLRLRSTEDAGASRFPTHLTHLQEPFHMPADVLGGLAPPHAVRAGADVAGTSGGPLRRLASSPPVQPRQGRRRRESGQRETVMVPAQRTGNTELPPDDGYTWRKYGQKDILGSRYPRSYYRCTHKNYYGCDAKKKVQRLDDDPFTYEVTYCGNHSCLTSTIPLFTLPDATATATTSSPIAATGSGLAPDDLRMAPAEQAHSAALSTSIHLGMSWMPASLQSIQAGTGSGAGVGSSGSAQMNVSTAGKDTDYPVLDLADVMFNSGCSGGSSMDGIFSAHDRRDF |
| >DgWRKY85.1 | MADGKPEAPPEDKHPALKAEVQKPPPPEAPDAALVAEAKPEKTVVTETEARKQEPREKPEPKEVKVKVEKKEKEKVEIEATRRPAGASADAPILAVPMLAVPCFIAPPGFAGHFAMSHQAALASVTAQAHMQLQSPTSSAYSEGLPSPYPHPITPKAIRPLQQASSLTQGSVGTPIAQSPSSSESKLHRHAAVNMVGDGFNWRKYGQKQVKSSDNSRSYYRCTHSSCSAKKKVEHCPDGRVIEIIYKGTHSHERPQKTRFVKEKVHHINVSPRGNETLRLVNTDIMESRTPTSKLNKSAVIEKSEQQLFCSSDCEGDDGNKSEDEHPTAEPQPKRRIIEATTPNLTPVLRTVREQKIIVQAGKTSDGYRWRKYGQKIVKGNPNPRSYYRCTHDGCPVRKHVEKAPDDINNMVVTYEGKHNHDQPFRSSNELRDGSVSAVTVAITTTEQPSTLSSTSDQKSPTSTQKAADSESAKDTSLELGGKKPPEGAQTMLSIKTNPDDMKSSLLKDTSAIVHVQNN |
| >DgWRKY45.1 | MQAELSRMNEENQRLRGMLTQVTNSYQALQMHLVALMQQRTQQMPPTQPQQPPGHEDGKNEGAMVPRQFLGLGPSGAGAGGDAAEEPSNSSTEVGSPRRSSSNGNEDTERGDNPEGPSTAGWLPGRAMSQQLAKGHDQQAQEATMRKARVSVRARSEAPIIADGCQWRKYGQKMAKGNPCPRAYYRCTMATGCPVRKQVQRCAEDRSILITTYEGTHNHPLPPAAMAMASTTSAAASMLLSGSMPSADGQGLMSSNFLARTVLPCSSSMATISASAPFPTVTLDLTHGPHGPPNALPLSAARPPAPGQFQIPLPGGGMAPAFAMPPHVLYSQSKFSGLQMSSDSSADAAAAQFAQPRPQMGLPGQLSDTVSAAAAAITADPNFTVALAAAITSIIGGQQAAAAGNSNANNNSNHNVTTTSNNTTSNNTNSETQ |
| >DgWRKY50.1 | MNAGSPDIRLSKREGRGVWSCLMEGSSSSSDRYTERCALATELAQVLDTVRQLEAHMGVKGGADDGGERCRALVSSMRSSVDRSIHIAMASCCAPESPPSAEGSPRSGGSDQAADSLCGRANAAGQCKKRKALPKWSTQVRVNSVQDVAPLDDGFSWRKYGQKDILGAKYPRAYFRCTYRHTQSCHASKQVQRADGDPLLFDVVYHGNHTCAQCSSQRAQHAASGKHCWQPQPAGAGGQERSSIVSVGLKAEGVANGLSETPFLFQSKPAGAADTSGDFPAAGCALTASPFVSPATSDCQLIRNVPDVELASATDSQMADMDFMLQLADADLFDNSRYF |
| >DgWRKY10.1 | MVFLLKAFVERFHAEMNGHTHTAMEWKDTSPAGPDSLLPSYLTDPFPSDPLVEDCTGTNEGSHAGFERHGLSVDVGSPQEEGKPMPATPQFGQRSGSSSSLSERMQARAGFSVGKLSMPCVTPAAADSEHSGGAQSPYLTIPAGLSPALLLESPVFLSNAMGQASPTTGKLFMLGDSNNNNLARLEAPTIEDGPGTFCFKSLDLRSSQYAAEGKKEFLPNSQHPPLPSRDASVKTETNIQTARDANSMGHLNQEQFNNAQDLMKSSYHDSNNNKCNRLAPDRAMASGGDKVSPPDHGPPVAMDSEAAKGDYPAVSAAMPAEDGYSWRKYGQKQVKHSEYPRSYYKCTYPNCQVKKKVERSHEGHVTEIIYKGGHDHPMPAAAGRRPAGVPQQAQPFGDAQMDAVDNNIGNGNSNAGASQQPNADARQLWHNGAGVQDWRGDGLEATSSPSVPGELCDSSASMQVHDGAARFESPEGVDVTSAVSDEVDGDDRVAHGSMFQVQGPVVDTEGDELESKRRKLESCAIEMSTASRAIREPRIVIQTTSEIDILDDGYRWRKYGQKVVKGNPNPRSYYKCTHPGCSVRKHVERASHDLKSVITTYEGKHNHEVPVARNGGHASSAAASGASQVIHAGGARRMEPQSLQDGLMRLGGCGAPYGLPPRDPLGPMSNFPYSLGGQAAALPSLPMPTGLGAVEGMKLPMLSPSLHPLFRQRQAMETAGFRVPKGEAKDETVPSNVAGAAGGAAYQQMMSRLPLGHRM |
| >DgWRKY32.1 | MEEVEVANRAAVESCHSVLALLSQQQDPALLKSIASETGEACAKFRKVVSLLSNGGFGSVGGAGHARSRLSRRRKPMGFLNQKGFLENSTNTPFGMLMVGSSTATPSPSAGSAAQSRPQFVGPPPDLRGLDLVSSGSKTAHQFGGPPKMVQPLSVQFQFGATAHRFPFQQQHQQKLQAQMFKRSNSAISLKFDSPSGGTGTISSPRSFMSSLSMDGSVASLDGKPPMHLICGPAVSDPLNARHGGPKRRCTGRGEDGSGKCAASGKCHCSKRRKLRIKRSIKVPAISNKISDIPPDEYSWRKYGQKPIKGSPHPRGYYKCSTVRGCPARKHVERCVDEPEMLIVTYEGEHNHNRLPTQSAQT |
| >DgWRKY82.1 | MTRTRAPFLPTSRRPRHALTSRTRHAPSRLFPRYFPPWTTLLAKAPEHYSIFLPVKKPQLRPSFRVNEPHHGRYEPLVQRWWTPIRATRRTRFENQTNEPSPQSSNAPVSTATRVDTHYVVRASTVCVHQYMSMAPPPPPFAQVVDDLIKGQHFAAQLQDLLRASPKAGLIMDQILHTFSRAIHAAEAAAAATSAGEWSSDVQSEVTDGGSGGGKRKSVAGAGDRRACRRKTQQSSVVTKTLKSLDDGQSWRKYGQKEIHNCKHPKAYFRCTHKFDQHCAAQRQVQRSDEDSDTYRVTYIGVHTCQGPAVASQIHHRTGTGGDVLHAGCHFINFAPNAVATPAATTSTTTTLTASTSNQLAGMDAATGSGLQRLKLEGGDQEEVLSCNTPGSSALRSTAGAAAAPTWPDLGDVTSTRQYGGDVDLGDQLFDDYSIYLDVLEDIVPYDFDH |
| >DgWRKY7.1 | MCDYFLQRMEGDQAGGGGDLTDIVRAGGAMPGNSDGDGGNLPSTAAEWQLQPAGQPMLFPHPPSSSDGADVFGDPFAGLGDPFSTDYSSGAANFLDGMPDAMAKVSFDSGMSACGGGRGGEQMLDMGRKPLLPRGMQQMMPGGIGGGMGPRLMPSPLSPIAIRPYPAMTAGDMMKLGITAGQAAGCAIDAAVAGMQMSSPRNSGGIKRRKNQARKVVCIPAPTTAGSRPTGEVVPSDLWAWRKYGQKPIKGSPHPRGYYRCSSSKGCSARKQVERSRTDPNMLVITYTSEHNHPWPTQRNALAGSTRSHHGKNGGSGSKSSHNEKQPQPNNVKEERKDHQTATTSAVTTTTSTSPMIVKEETTTLARSSDQSLARDQRSMDTAAGVQQVDHSDHVFTESYRPMIAESSHHEDFFADFADLTELESDPMSLFFSKEYMEARPSGIGDHAQEKAAVAKELDPFDMLDWSTTSTAGSTFEQGKRS |
| >DgWRKY89.1 | MEGGAGWYYRSGGSNDWDLNAVVRYACRGRVSPPMVPDDPFSSFFLPPPPPQEATGDGLPDVATLLADLPFNGDAAAVDELSIAFFGPSAPPAPPQQQEPAVATIEETQPLQQDHVAPPPMPQTSGRQASGGDGSSRSKRKKKQVKKEVKRVAVDGVSADPWAWRKYGQKPIKGSPYPRGYYRCSTDKACEARKMVERCRDDPDTFILTYTGGDHNHDAPTHRNSLAGTTRNRQQQHREPPAGGQLGAPGGATATARAEPSPGQSTSGGTSAASPTTTTSPRSPSTEECNQEEAECDDAGGASMVPKDVKMEPEEDDELKKLLDTAIGVGGDSSRYMRMDDDGGGVGVSSLLNAVEETFAVTPWLTSLGDATGWS |
| >DgWRKY44.1 | MAASLGLSSSSHEAYYYQAADPLAAAAADSAAQLGFSELLGAVRPPGYSPAPPGYFAAGGETMNSFPATSYYCSGAGAYDGGGAGRGASGRPSSAGRIGFRTRSEVEVMDDGFRWRKYGKKAVKSSPNLRNYYRCSAEGCGVKKRVERDRDDPRYVITSYDGVHNHATPGSYHGASASREAAYSAPAPPTWIWSDLHAAAAAAAHSSESSY |
| >DgWRKY58.1 | MALSTPTAVVLELMTMGQQSAAHLGELLAAATPPTEAELGQALTAEILRCCDRVIAAISRASVGTKRKAIVERGAAMPSKRRARGAEAHREVQSGTTADGFVWRKYGQKDINGSKHPRFYYRCAYSGEGCGATRRVQQSQEDPMAFVISYYGDHTCGGGAGDPCHGEPQAAMPPAVVYSGLNAGGVVGVLDRYQNMEVESPQPLLAAEQSWCRHGEAPGETSRGLSPSSSSSSSSEAELGASPVLEFLEGSLGVGWESVINYLGFADLPQTAMPQNFGDLPQTAMPQYFGDLPQTAWPQ |
| >DgWRKY62.1 | MGSPRPKGESFDFEEAMGSASASYSPPGSVFGLSPSDSSPRDIRKRRKDRPSWVKHTFTPHFDGHLWRKYGQKNIKDSAFPRLYYRCSYREDKQCLASKLVQQENNNDTPLFRVTYTYEHTCNAAPVPTPDVVAELPAPAGDSLFLRFDSTAISNGGDMHRMEQERQYQQSMAPGWPSLMLSFDSNRQPHTEHAAFPSELPPMASSSPPFSTDGAMWRAPPLPSPSMTTDSGGERFLTSDSLRYGLNDHVHFGDNGYLPDNGNEDNY |
| >DgWRKY60.1 | MQTQSRLVVNANGGAGTGFDSAAAADEQYHEAVARELTRGQELTAQLQAEALRALRGQGQAEATAAFILREVSRAFNVCISIMGGSAPAADAPPHATVAADAAAGARRARDDGVPRKRIMTPWPNSDGYQWRKYGQKRIMRTSFPRCYYRCSYHRERSCPATKQVQEQHSSNGSPRTFLVIYGHEHTCDLSSPAAAEPETARSPVDPIDFSAGLSRQQPGGVVQLSKEELEQQVLVSSLACVLQGSQQCYSGGGSPEEWLSPGRVGEDGAPAPAVSIETSTELDVMDYDVTDTVYFGASSSYGGDDGMLLNLRGA |
| >DgWRKY57.1 | MTTSSSGSVETSANSRPGSFSFANANFTDILGSSTATGSASGYKSMTPPSLPLSPSLMSPSSYFNMPAGMNLADFLDSPVLLTSSIFPSPTTGAFGSQQFNWRPEAPPASAAEQGVKEEQRQQPYSDFSFQTAPASNEEATQATTTTFQPPAPQVRSQTGEETYRGQQQQQPWGYQQQPAGGMEVGANPASFSAPAVQATSSEMAPSGGVYRQTHSQRRSSDDGYNWRKYGQKQVKGSENPRSYYKCTFPNCPTKKKVETSIEGQITEIVYKGTHNHAKPLNTRRGSGGGAAAAQVLQSGGGDASEHSYGAMSGAPVSTPENSSASFGDDEIGASSPRAGNVGGDDLDDDEPDSKKWRKDGDSEGMNNIAGNRTVREPRVVVQTMSDIDILDDGYRWRKYGQKVVKGNPNPRSYYKCTTVGCPVRKHVERASHDLRAVITTYEGKHNHDVPAARGSAALYRPAPRAAAEGAMSTSHQYTANQQAPPAMAYQASAAAATQQYAPRPDGFGAQNQGSFGFNGGSFGFSAGFDNPTGSYMSQHQQQQRQNDAMHASGAKEEPREDMFFQHSQF |
| >DgWRKY61.1 | MAFVQETVEQLWQELVGGYNLNARLLALLEHPMDIRGQELSRVFMVSLFTLKPGHRSRVAEVRMMAPEAMITEGSVGQRTPGTDKLICGEEVVPHRKRIREGIIKKEITASPHMEGYRWKKYGQKNIQNRKFPRLYYKCMYSHERGCRAKKRVQQQDNTSGAHRPVFQVTFVNEHTCHQVLPSQNISNNTTANTTTTMTSDGAHFDPAVDVGDNAGLQNNVMTCALATVIGGAPSQPPPVEVNQLSDSASYMSPRLPEVSMDFSCGSLFPPPVETPAAPSSSSLSPLRPVEAGSSYPVDGENIPSMDLMTMDEMFCHFSCGPLFSPIAAQSSISCWDDVPMAVVAGRYTDTASPWPHYS |
| >DgWRKY59.1 | MALSTPTAVVLQLMTMGQQSAAHLGELLAAASPPMEAELGQALTAEILRCCDRVIAAVSRGAAGTKRKAVTEHRAAMPAKRRARGAEAHREVQSGTTADGFVWRKYGQKDINGSKHPRFYYRCAYSGEGCGATRRVQQSQEDPAAFVIAYYGDHTCGGGAGDACQREARAAMPPAVIYSGLNAGGVVDRYQNVVSPPPLAAEQSWRRHGQAPDEASRGQWSSSSSSSSESELGPSPVLEFLEGSLGVGWGCAVNYLGFAGDLPEFSFGGTST |
| >DgWRKY63.1 | MAKSESNMVSSSGCSNGEHKRLQQHDSRDGGYAQQQAKKKARIGMKTDYTYAPYHDGFQWRKYGQKMIRGNAFPRCYYRCTYHQDLGCLASKHVEQHNSGDPPLFRVVYTNDHTCSGGDAAGSDYMASSMQIQQIADASLRKVPEMEIKPRPLTQLARCGRGFAAAIKEEKDAIVSSLLTVIRGCDDAVAKSDHSVRVKYDDSLLASNCYAGSPAVAGGSHEGSSSSSVSPVVLPAHDDLGLDFMVESNWFEPLDLGWFIE |
| >DgWRKY46.1 | MAFGQDAVEQLSRELLGGYNLNTRLLALLGGGPLDGRRQEAAVAMIQELSGVFRNLMVSLFMLNSPPEAKITPAKYKRISRGPHREVRGNVVREEITASPLMDAYKWRKYGQKNIHNREFARVYYRCMYSHGRGCRAKKQVQQRDNSSRRPMYQVTYVNQHTCHQVLPNEPTRATPTLTPKENNSTAMSGHFDHPHHHIVDGNAEFENEIMTSTLTTVIGGAPSTSSLTLPPVLESSQPRDLASTYMPGGAHLPSQVDANLDLDETTFVLRRSPLPPVKAPAAPWSPPLPVQVGSSSNSGSASAVGGYWSTLDPMIIEEIFQQFSPVAAQSISQYCWDDMPPMAVIRFPDSTSAWPQFYS |
| >DgWRKY86.1 | MAVDLIGRGYPPRSLAAEEQHEQLAFQEAAAAGLRSLELLVSSLSPRADRATPLGEIADQAVSRFRRVISILDRTGHARFRRGPVGGAAAALTSPSVSSPPPPMPVRAPAPPPASSLQLASQKSLTLDFTKPSKAPAAVAAAAPSVTSTSFFSSVTAGGDGSVSKGRSQLVSSGKPPLAAGTKRKQLPPPCASGAHSDAGGGRCHCSKKRKHRARYTMRVPAVSSRTADIPGDEYSWRKYGQKPIKGSPYPRGYYRCSTVKGCPARKHVERATDDPATLVVTYEGDHCHDASPPAANAN |
| >DgWRKY84.1 | MEAAPICSLADAMFRLPAKLHRLLTSHGHTLPRGAQDEIPLIKQDLEKILVFLQGHDDLEAEDCGMIVKCLTKEVRELSYDMEDSIDQYEHAADSWKRRGTLSPYRKKYKITRRMSKNSSRLQENLKWRLWMANKIREFSMRSQEALQRCSLFDLGSISSSTAATSTRCTTSFGSWHPKLYREPVGIGAPMKKLEAWLSKDGEQKLKVVSLVGCGGVGKTTLANELYRKIGGQFECQAFVRTPRKPDIRRLLISMLSQVRPHQPPQTCKVHSLITEIKTHLQDKRYLIVIDDVCATETWDMVTRALPDGSLRSGVLITTEMEDVALKCCCYDSKYVFAMKPLCHNDSRQLFFDTAFGPGYECPPELSEVANNILRKCNGLPLATVSVAGLLVSQMDKPGRLDCVYKSLCHGLRTNPTSDWMKQVLNFSYNNLPQHLKVCVMYLSIYQEDYIVQKDDLVNLWIAEGFIRATEEKDKEEISRGYFDELIFRRLIQPVQINDKGDILSCSVHQLVLDFVAQKSIEENFVTAIDHCQTTARLSDKVRRLSLHFGNAEAASPTNMRLSQVRTLAFWGVFKCMPSIVEFGLLQVLILHLWSDDESIGFDLTRISELFRLRYFHVTCNATLQVPPTQMRGLQYLETLKIDARVSAVPSDIVHLPGLLRLSIPVKTNLPNCIGHMTSLRTLGYFDVSVNSIENMQSLGELTSLQDLRLTCSTVPSTYPKSNMDYMSSILAKLSNLRSLILEPSSNLDVKSSSMSISCDTLSIVSSPPSLLQRFEWLPRICTFSSLPKWIGHLDKLCILKIGVSKLASNDVGLLRGLPALTVLSLYVRTEPAKRIVFDKTGFSVLKYFKFRCSVPWLEFEVGAMPNLQRLKLGFDARGTDQHVSIPVGVQHLSCLKEISVKIGGAGADDPDRNRTAAESALSCAIKMHPAHPTINIQHVDLMFTRKDDYNSSFKEEEHMALQKQYEIMEEDSIEQHQVLQKDFREDASIIVPHRKKRLKWNTQVKVSSWQDAEGADGYIWRKYGQKDIIGSKYPSSCRGYYRCKYRHSQGCLATKQLQSTDGDPLLFDATYNGNHTCRQGENLHLQLGHEQSSVSVGVKAQESAHGLEHMPPR |
| >DgWRKY14.1 | MEEEHCGNNWDLHAVVRFGCRRALSSTPPPRDDPFAPFLPPPTPVKEQPADAGWCFPDLGAGFGQDADELLKAFCAASPPTQLPLPPPTTPPPPPPQQQQQEMMAPAEVLLQTAAPAPRAQPSGRQASGGVPRSKRRKNQQKKVVCHVAADGVSSDVWAWRKYGQKPIKGSPYPRGYYRCSTAKGCPARKQLERSRADPNTFILTFTGDHNHAAPTHRNSLAGTSRNKFPSTSSSARPRPPPPSVVVAGEPHPSTSAGLSPTTPLRTSSSSEEEDELLVEDMEMAGEDDLLFLSGDSDNAATPLSSLFDVDEPFLGSHWTGMATGAAGAAGS |
| >DgWRKY64.1 | MGDVPRSQAVADEDEVGVWPGELDEQLISELLSDDSLLAALVPANDSENHSRGSGIAAAAPCDSDGGGTAAEHEQLPPASAGSHAMCSVYSGPTIRDIEKALWSRPYPSGRRYGSLYLSRRYGEPSTAPESKKTIKVRSCGSKTPADGYKWRKYGQKCIKNNPHPRSYYKCTSSHCSAKKHVEKSTDDPEMLIVTYEGAHLHGPQPLLRRLQPPAATAADQPDAAGDAVSETLAKRKKASKPSPPASEGYHGAADGIRPSNGMSDEEVGGGSKPQGRQMGRSHDAVQPVATMFSSSESPPSSWSGREDFNWSLEALLPVQRI |
| >DgWRKY47.1 | MELRPKQHHHSRNNDEDAMDDSGHLSLRTGTGRRSDDEVGRRRGEIREVDFFSASGDPGTRSRKADSGGCRVTHGCLDDEVHTGLDLMTTARCAAAPATVDEERAGTEKNNKMEAATVAVEAELRRVLEENLRLRGMLEELTRSYGVLYHQLHQVTHQQGHPHRHHPDVLMNNQLPPTLPNLTRIGAPNTSMAQQDDEMIAADSPSFSSGGKRRTGQDEAAPPGTRERSSEQASSEQLPCRKPRVSVRARSEAPMISDGCQWRKYGQKMAKGNPCPRAYYRCTMAVGCPVRKQVQRCAEDKTVLVTTYEGNHNHQLPPAATTMANTTSAAAAMLLSGPATSRDGAALFGHPTVFHHHQSFPYASTVATLSASAPFPTITLDLTQAPAGANGMPHRLPSVQQQPQAMPFAAPPHLAMYLQQRASRVLPARPGPDLDARQQSMMETVTAAIAADPNFSTALAAAISSVMAGEAHRQDHPPIPRGSAVGQVHGDGAAGVGTAAPTDGSHVPASGGSPRLGTQSCTTSTT |
| >DgWRKY66.1 | MTKSEVDHLEDGYRWRKYGQKAVKNSPFPRSYYRCTTQKCPVKKRVERSYQDAAVVITTYEGKHIHPIPSTLRGSAAHHLLAAHAHGLHYPHFPMPAPHPAPGSAFRPDGALASFLQQQQQLQGGGGHHAAMQQAMQHQQQLAAAAESTMRQANAAAAAMGANSSASAGATATGGAPLRMEHFMSQDYGLLQDMLMPSSFAHSDGTATNSHNSHNRR |
| >DgWRKY72.1 | MAAVGAAPVLYQQAAADASCFFSSSSSSSISSYFSHGHGAVSSTTSSSPASSFSAGLGASPVPSPPQPAIADNAAQFDISEYLFAELPLNDLLAPMQQDGATGSRGIPGANATTHTHARRAAVPAAMARPRTERIAFRTRSEIEILDDGYKWRKYGKKSVKNSPNPRNYYRCSTEGCNVKKRVERDRDDTAYVVTTYEGTHSHVSPSRLLRHPGRRLRPLLRCRHPPGTRLARLTISTLPHWRAYVEISTGLADLLPGRCGAAGEKLR |
| >DgWRKY73.1 | MDNFHEGEEDHLQLQGDDDLLRHLQYLAPPPLMDQQQQQQTSSAIPELVDWASLLLPLGPGAAGTSQQVPAAGVGEDGMVGGGTVAAETGGSSSAGAGVSGGEQVIKERKATGARGRKASRPRFAFQTKSENDVLDDGYRWRKYGQKAVKNSAFPRSYYRCTHHTCNVKKQVQRLAKDTSIVVTTYEGVHNHPCEKLMEALSPILRQLQFLSQL |
| >DgWRKY34.1 | MALDSVQSYPSDLGSNSRATRQQRFRKEEQTWTADTYAPYDDGHQWRKYGEKKLSNSNFPRFYYRCTYKTDMKCPATKQVQQKDMSDPPLFTVTYFNHHSCSTTSRPIGSAPDTPEQPSSRKAVSICFGSQASEQPTFLTSPSALQSPASRASQQQDTGTYARQFQWTDTSPSAGDDAPVKMEADDLSGTSSSSVISGGALPRTLLPIGQSRCIEYFQFL |
| >DgWRKY37.1 | MEGVEESNREAVQSCHRVLNLLSNPHGQLVPHKELVDATGEAVSKFGSVATKIAANGNGRQGHARVRRRIKKPMPMFDSSLFLESSASAADAAAAKASQPGPDTSLRLFPRYQQVEGSSSKEPVRIPAQFPRRLLLENPSVGSNGPARGPPVQLVQPVSVAPPAGTPAPALPAAHLHFLQQQQSYQRFQLMHQMKLQSEMVKRGGLGEQGGSNGGVNLKFASSNCTGSSSRSFLSSLSMEGSMASLDGSRSGRPFQLVSGSQTSSTPELGLMQRKRCAGREDGSGRCATGGRCHCAKKRKLRIRRSIKVPAISNKVADIPADEFSWRKYGQKPIKGSPHPRGYYKCSSVRGCPARKHVERCVDDPAMLIVTYEGDHNHNRAAAQPA |
| >DgWRKY36.1 | MAGAAGDRPEEVGGGGGDWPFAGDAFPEYSSAMFAELGGWPGGLGTGAGDLKPLDLSAGTPSPAPVSVALTSEGTPSRSRSADAGGASSSSSGDGVGADTGKLAAAADVAASMKPAVKKGQQRARQQRFAFMTKSEIDHLEDGYRWRKYGQKAVKNSPYPRSYYKCTNSKCTVKKRVERSSEDRSVVITTYEGQHCHHTTSFQRGATAMQFHGAATISLAEQMSFVSAQQLYNLPPLRRQVNPTLSESVVSSPSASFGQQLNGSDEPPRPSIGYSPMVSMVQSPSSSMVPPAVSFDAGLLGDMVPPGVRNG |
| >DgWRKY35.1 | MGDRRGDEGMRQQPPFSSGHQERVFDGGGPAPYGSDYDPGSSYMSLLGTSMSNPQPPPWAVEEVTASTPINLTPQFSMANYAPSSSYQQQNASFASPLGANLQHYSSSSSYFHADPPPQWPPRATQPPSSSLLLPRNFSVHHTAPPTHYEQHMQLLRAAALGGQQAAPAAPPIEQPAKDGYNWRKYGQKQLKDAESPRSYYKCTREGCPVKKIVERSFDGCIKEITYKGRHTHPRPPEPRRGGQAGSDDLAAAAAAAAGAEEDEASDDDMLHDDDGEEGHDIGKGAGGAAGQRVVKKHKIILQTPSEVDLLDDGYRWRKYGQKVVKGNPRPRSYYKCTAENCNVRKQIERASTDPRCVLTTYTGRHSHDPPGRGAGATTGGGGSSSDPTVNPPGGTLHQSSGTHRLKEESRD |
| >DgWRKY17.1 | MTSSGGGGGDVPAERAAAAVNDLREAQEGAERLRGWLQEQSSERTEQMDVILNKLMSALSALDTGGAAGASATGSGDGVLRPTAESSSRRTRKRSFSRRSQRSSGTRVTHTLIDGHIWRKYGQKDIQNSDHARSYYRCTHKSDRGCDAKRQVQVCETDPSKYAITYYGEHTCTMATATAPMIIVDGNDGRGRNNLVSFAQTFPQLAEKEGGGAATTRQLSSSWCTSDGVFSSSGVDQFVQTDELAAVVGSAGRTSSTVGSVPDYGGLGDMAGGGAQGCGTSSFPSSPSSLEFMVNSLSSIVGDDDLFRCDP |
| >DgWRKY29.1 | MTGTSDRGSIMEDWMAMPPTPSPRTLMSNFLNEEFSSGPFSNLFGEHGSNKPQGQSEKRGELVDLREQVPAQSAQATPRKDISLQPNFFNANQKSNPHGGLAERMASRAGFSIPKIDTSRVGSSTVVRSPISIPPGLSPTTLLESPVFLFNNMAQPSPTTGKLPFLMATEANSAMPRAGKMNDDSAFSNDVFSFQPHLESKVPSFPYVEKGYIACNQNQSLSNMHQQESSLQSSFTATKDTADETIVQPKTSDSMFGDNHSSEEQEDDETDQNGEYSSATISTPAEDGYNWRKYGQKQVKSCEHPRSYYKCTHPNCPVKKKVERSEDGQITEIIYKGSHEHPLPPPNRRPGVPLSHINDPQVHVLERPGSHEGLNSASLWGNGKSGCIHDVQNEGIEGRPSASPHVSAYGDTSTVESQDAVDVSSTLSNEEMDRATHGTVSIDCDGGEDETEYKRRKLDALAAPTLPTVNTTSTIDMVAAASRAVREPRVVVQTTSEVDILDDGYRWRKYGQKVVKGNPNPRSYYKCTHAGCSVRKHVERASHDLKSVITTYEGKHNHEVPAARNSGHASSGSGNAPPSAPQASLSHRRQEQSQGTFAQFGGASPFGSFGIPPGGQLGAAPGNFRFGMAPPGMSMPMPIARHPSMMQGFPGLMMPEGQPKAEPGTQSGFPAANAAYQQMMMSRPPFGPQM |
| >DgWRKY79.1 | MCDLFWPSPGEQGDLSDIVRASLHPPHHHQLPTAPASPYLQPEKEEEYNSLLLEGGSGGGGLVVSHGDDEQLGMVAMMMMGDNTRATSYHHAVSLHSPRTAVYPQASQEPLPGMIRQPDIEREGEVVVAPEIGDRLQHMSIVHHPRVPAAMKPRKSQMKKVVCIPAPTAAPGVSGRPSTTGEVVPSDLWAWRKYGQKPIKGSPYPRGYYRCSSSKGCPARKQVERSRTDPNMLVITYTSEHNHPWPTQRNALAGSTRPASSAAAKNSIPSSLAATAPGPTRDSIINVDCAGVNHQLKQENDLDMFADMDALNIFSSITKIHEDDSKEQPFDPFSFSDYL |
| >DgWRKY53.1 | MAVAGAAGTAYRYLPHGAAADVCRSMAFPTPRGGGSGSGSSLPENSNPYGSSSAASSFSPAFAAVARGALQQQLDVLDYLSDDGGLAPSPEVPETLRAPLPPRMPVEAAAPVVPDAGYAAHHPRRSAPSVAAGNKIAFRTRSEDEDVLDDGYKWRKYGKKSVKNSPSPRNYFRCSTEGCSVKKRVERDKDDMNYVLTMYEGVHNHPSPGTVYYASRDPSTGRFFVAGTHSLGP |
| >DgWRKY52.1 | MAVAGAAGTAYRYCPYGAAANVCRSMAFPTPRGGGGSGSSLPENSRPYGSGSAASSFSPAFAAAASGALQQQLDVLDYLSDDGGLAPSPEVPETFPAPLPPLMPVEAAPVVPDVGHAAHHPRRSAPSAAAGNKIAFRTRSEDEEVLDDGYKWRKYGKKSVKNSPSPRNYYRCSTEGGSVKKRVERDEDDMNYVLTMYEGVHNHQRSGTVYYASQDPSTSRFFVAGTHSLGP |
| >DgWRKY31.1 | MMKGEMQLGSHEERLKGKMKEDHRASDDNFLKSLRNQSSTKEEVLQDKIAATRVEMVEVRKENERLKTTLSRMIEGHRSLQKQFDVLHQGRSKNSPDHDLPADIEEPGFVSLTLGTSTSRYNMEEKSSDNSEAGKGIEGSLKIRESGISLGLSDKRVGSATDCSETKVQPNVLILSPEGSSDEAAKEDAVETAQWPPSKTLKSSRSVGSGEIDDAIAPQTMAKKARVSVRARCDTPTMNDGCQWRKYGQKISKGNPCPRAYYRCTVATGCPVRKQVQRCSDDMSILITTYEGAHNHPLSASAAAMASTTSAAASMLTSGSSTSLDSFPTPGLSFGLSAPAANVSNQHRPFFLPAAAAASITATPSYPTITLDLTSPAQAFSLSNRFSSSFAGPSHSTTSRYPSTSFSFSGSGGPSSLTGATAWPASAGYLSYGSSSGVSYNGAGKSPFESGLSSIHGRQGSTSSLYQPVQQRAVSVSRGSTGMAAPSGVITDTIAKAISADPGFHTALAAAITSYVGKPAGGSSKGLEWGEHLGLGPSSGAAACSSGLLSRSSSSSMAAQSSSSGQMFFKPSVAVSGSTSTASASPVKNREHTN |
| >DgWRKY6.1 | MEAAVVSVSEGAIGSLLEKLGDLLAGNYKLLKEAVDDIMFLKAELESMRAFLERMSEAEEEPDKQAKCWANEVRDLSYDIEDSVDEFMRPVECESNSKPSGFFKGFIQRSMNLLTTMNSQHKIAKEFRGLKRRIMEVSERRTRYKIDDDVFKPNNISIDIRMLALYEDTTGLVGIDVLRDELIKRMVGEESVSTQQMKVLSIMGFGGLGKTTLANQIYDKLMCQFECGAFVSISQKPNIRNIFRRMLSQVGYVAPEGTNMDIWVEDELISALREFLMDKRYFIVIDDVWDETAWNVIRCALPRNCKGSMVITTTRIDTVARACCCNQRDYVYKMKSLSHEDSRKLFFERVFGSEDACPLYLEEVSAQILKRCGGLPLAIVTISSLLASERTKLKEHWEHIMDSLAPNFQVKPTLEGMRQILNLSYINLPRYLKTCMLYLCIYPEDYIIKKNDLARQWVAQGFVSKAHGRNPENVAGGYFNELINRSIIQPVDTDHDNEVLSCRLHDMMLDLIIHKCREENFITATDDIQAMIGLPDKVRRLSLNLDGIIDGTILETIRLSQVRALARFGTSVYAPPLLKFKHLRVLALEFYSGNRQEIIDLTGMSHLFQLRYLKIKADCKIVLPSNIRRLEHLETLELGYDLEVPSDVIHLQGLLHLIIPNGTNLPGGIGNMKSLCTLHAFDVGLNSIDNIRDLGNLTNLRDLQICFAKLEGLDDMERRQRLDVLRCSLEKLCNLRYLLTDSKISSTYALNPLSAFPRLLRRLQRLWVFSRVPQWIGELHNLFDLDLEVVVLENDIGILAQLQSLNHLRLHVKGTPEAEDKLIIYGTGFPVLKYFRLSSTKISQLTFEAGAMPSLQELVIILDSQYGAQPMGIEHLLSLKKILVQFLGRHFDDGFSWRKYGQKEVRGAKHPRSYFRCTYKFSLGCKAQRQVRRTDDDPELFHLTYIGKHSYNQCTIPLLQPGQEQSVEVQQLI |
| >DgWRKY8.1 | MEGASQLEVCLPSLYALDPYTAPPPLLAPLPNQQKLIQMPLVHGQSGNHGVLFSSDHGGGLYPLLPGIPFVHSIPAAPVCEKSTGFASLGGAGEAGTSAATASNEVASTTTATAATCHGASSWWKGTEKGKMKVRRKMREPRFCFQTRSEVDVLDDGYKWRKYGQKVVKNSLHPRSYYRCTHTNCRVKKRVERLSEDCRMVITTYEGRHTHIPCSDDDDAGDHTGSCAFTSF |
| >DgWRKY39.1 | MEEVEEANRIAVESCHRVLGLLSQPQDPAQLRSIALGTDDACAKFRKVVSLLSNEGGVSHPRAKVATRKQTPGFLSQKGFLDNNTPVVVLNSSAHPSTSSAQVYPRNSILDSHQIGGPPKLVQPLSAHFQFGNVSRYQFQHQHQQQKMQAEMFKRSNSISGINLKFDTPGAAGSMSSARSFMSSLSMDGSVASMDAKSSSFHLIGGPAMSDPVNAQQAPRKRCTGRGEDGNGKCAVNGRCHCSKRSRKLRVKRTIKVPAISNKIADIPPDEYSWRKYGQKPIKGSPHPRGYYKCSSMRGCPARKHVERCVDDASMLIVTYEGDHNHTRMPAQSAQA |
| >DgWRKY56.1 | MDGEWSDGAVSGGEQKARGDGVSADSIPGSPPAPPSTATSSAPSGRRRSLQKRVVTVPLADVSVPRPKGVGEGNTPTDSWAWRKYGQKPIKGSPFPRAYYRCSSSKGCPARKQVERSQADPDMVLVTYSYEHNHSSAVARTQHRPPQKPQNREQPVPPPDQPADQSDHTQQGAAVHQLVTESSPAPGIEVHDEFRWLYDVVSVTSSTSPSDVEEADDMLLYGPMFFGKAAVGTASLLPDEFGDMLGGEGTTSEEDAMFAGLGELPECAMVFRRNGLPGGVKVEQPAEGTAMT |
| >DgWRKY55.1 | MQQRDMDDLHGDDDEAHTFPCFPYFAVPSTPRLPSLLASSDDQHSPLAAALQLPSPPWPCNPTDLNPQTMLPAMVDWSSLFQTGMAAPSEQQQEEAVQADQRVENDCEAVGSGNYNMVNVKGAVGRSGKKKASKPRFAFQTRSENDILDDGYRWRKSYYRCTHPTCNVKKQVQRLAKDTGIVVTTYEGAHNHPCEKLMEALGPILKQLQFLSQL |
| >DgWRKY54.1 | MSAEDGYCSTGTDSPRADSEPAADADSPGGGQSKRDHPSPSSTLPPPKRSRRSVEKRVVSVPISECGERARINGEGPPPPDSWAWRKYGQKPIKGSPYPRGYYRCSSSKGCPARKQVERSRTDPTVLLVTYSYDHNHPWPTPKGGGCHANKSSPRLAEPKPEPGTPADSSQHEPAEQETSEPVVPKQETSDPVVPKQELEDQEEEQEQKPDVVSLAAEPAAITASVTSVEDEEESFDFGWFDQYPTWHRTASLYVPAFDAAPPLLPPEEWERELQGEDTLFAGLGELPECAIVFGRRPEIALAATAPCS |
| >DgWRKY90.1 | MDEQWMIGQASPSLSLGLNLGRPTARREVTTKVLVEEDFMSVKKSHEVEALEAELRRVGEENRRLGEMLRALVAKYGELQGKVTGMMAAAAANQHQSSTTSEGGSAASPSRKRARSDSLDTAGGHRNPSPPLAAAGSSRFAVTVAAVGPDQTECTSVHETSKRVRADECKPSRVSKSYVHADPADLSLVVKDGYQWRKYGQKVTKDNPCPRAYFRCSFAPGCPVKKKVQRSADDITVLVATYEGEHNHGQPAAKHDAGKKSDTSAAAVRTSPPAPVVQQHHHQQQQQQQQQQNQAAAEAERQNLAEQMAATLTRDPGFKAALVSALSGRILELTPSNS |
| >DgWRKY78.1 | MAMRRPKSEMSPPASPSPSDHRDAVIEELQKGAQLAESLRQQVELIPELDRRHAALANVSDISTALASSLSVLQFEREQYSSPSSFSDAATAHAAGAFGGGGVRARNGAVARSRKAKHRRGADGQELPIKEMLTETPENDGFHWRKYGEKKILNATFPRLYYRCGYSEEHKCPAKKYVQQQNYRDPPEFMVTLIDDHTCHALFPAEADRQPPSSSSSVNSQLLDFTKASLSSAAGVSRMKEEDVAGGMSVGVSSYTYDELYSSSSLPLMSPKQWEMEMEIKSLYRRHSGDGSC |
| >DgWRKY77.1 | MAALFTPPASSVSNLMVQGRESAAALESLLRGASAQEHGEIQELAAEILRCCDRALAALHHGAVVDDGADGRKRKSGAAAQRRPNKRARASGRQAADKRVEKKWTAEDGFIWRKYGQKEILNSKHPRLYFRCTYRDDSGCTATRQVQMSEDDPSLYVITYFGNHTCCADVAAAGAFEVKDLGETHPFVINFGSAAAASSGSPLWLSSSSDDCCGRSEASRSFSQNGCLPEGGNGEEEFQSKVAKVEAASSSDVQPAAEPSSSGDASSSSPPWDPLAACSEWDIFNELETSFDYVSDFLQV |
| >DgWRKY41.1 | MDMEKQGDGAASAQEGDLADVVARANIAMASSSTSHHQPRPPPPASAHMMVPYEEERRQQPMSVASGVGVGNAAAMSAVVVDPYLLAATGYGLPQHQHQQLLDFQISEHAFCGAGVGGDVDAREDAMRISPPHAPPPHEMINRKNDVRKVVCIPAPPAMSNRAGGGGEVIPSDLWAWRKYGQKPIKGSPYPRGYYRCSSSKGCMARKQVERSRSDPNMLVITYTAEHNHPWPMHRNVLAGYARAQSTNSTTSTVKKQKITSITDNIASSSSSNHNNFHPVQNVMGHNNQTTSKSVDVAVAGDGSTGIREGGGLMYELSCNGIVQPDEVFAELEELEPDNSALMISANVYSRGVSSNYEWHKF |
| >DgWRKY12.1 | MSSSKRKREAIDLERRDDVEGGDRRADDGVPAAGKEGQVKPGEAPSEEVVEVVVDRGEDGSKEEIKYGTPQGEVMEEDKQSAADHDADEKSADDRVETRADDKHMVEETPGDGDGDGDGENHTAMAHDELSTMQEEMEKMKEENKMLRLVVDRTVREYYDLQTKLAAYQQQKQPADEPKEPEVFLSLGGTAPAAGSLIAEAKSKAEAAHRRPSVGSDETEDGGEGLGLSLSLGASSYDEEARHDSGAGLDVVSDGKERGYALLESSKMHAPAAAEDLAAAGIGTQGGVNPANRKTRVSVRVRCQGPTMNDGCQWRKYGQKVAKGNPCPRAYYRCTVAPACPVRKQVQRCQDDMSILITTYEGTHNHPLPVGATAMASTANSASAATFMLLSSTSSDAALSAGPPPSSSSSSSSYLSPYMQHNSTSHSHYHSSAAMAGSGAQHLNLFGHSSALAAQQASHLKYPWPPNPSQGGSAAGLGGGKRPFWGTGGVNDARPSAFPDNAGTVASEQNRFSAAVAAAISNLTGKDGQVTGGNKEGESSNKWGVVESLPPHD |
| >DgWRKY49.1 | MSSGGGGGGDQDLHGLYHQHGHGHLTRSDDASTGYQFSSNDMESFFFSQQPVGIGGSGGSADEIVPYSSMTDYLQGFLDPAGLVRHLDLPSQDVPTKHELSVDVRSHDRDSQGTGSAAGEGAALLTPKSSVSFSSSGGEGEGKSRRCMKGPAKEADEEDAKDQKDDEENVKKVNNKAAKKRAEKRQRLPRVSFLTKSEVDHLEDGYRWRKYGQKAVKNSPYPRSYYRCTTPKCGVKKRVERSYQDPSTVITTYEGQHTHHSPASLRGSAAHLFMPPGLHGMPPPHLMPPGTFHPDLMSMMHMQYTSPGMYLPPPLATPPAALQQHHITDYALLQDFFPSTMPNNNP |
| >DgWRKY25.1 | MESLVDVNGGGSRLVVTELSHIKELVKQLDVHLGGSPDLCKKLAAQIFTVTEKSISMIRSGHFDFGGRKRSAMGAGLDSPPFSSTPSPLSGVSDMAFKRSKKRKMDKGKHQVRVRSAGGPDAPEDDGFSWRKYGQKDILGAKNPRGYYRCTYQKSQGCAATKQVQRAEEDPTLYDVIYNGEHTCVHKSTAAAVAVAKSQPEDARSLLQSLSSSLKVETEGLTPRAQQGWSATTPFSFSSPAVSGLTPSTPENCFGQGLSVPTSLEPSPATSDSNHFSVRRAQSELQTMVSAFMTVSSMQQPAMEETAFSLDGIDFDIDNFDVSCFFASDC |
| >DgWRKY24.1 | MESTVDSNGGGSRLVMTELSHIRELVKQLDVHLGGCDDLCKHLAAQISTVTERSLNMIMSGHFDGRKRSAAGADIDSPPSSETPTPLSGVSRMPFYKENKKRKMMGKGKRQVRMRAAGAGADVPEDDGFSWRKYGQKEILGAQYPRAYYRCTHQKTQGCSAFKQVQRADEDPAFFDVTYHGTHTCGHKTAAAASVQQAAANPDAGCLLQSLRSTLTVNTAEGLMSGPQQGWSRTAPFSFSPAASGLTLPELFSTPSTPENCFGQGVSVSPSQLQLSPATSDTSNFPMDTFEAGSEYDQILSALVAAGSTQQPAMDVEEAAFSLDDLDFDISYFLVDLA |
| >DgWRKY23.1 | MESLVDGNGGGSRLVMTELSHIKELLKQLDVHLGGCPDLCKHLAAQIVTVTERSIGMIMSGHFHGPKRSAADAGIDSPSALGGVSGMPKKRKMMEKGERRVRETSINDGYSWRKYGQKEILGAQHPRGYYRCTYRKTQGCAATKQVQRADEDRTVYDVTYHGTHTCAHKTAAAANVQPAAPNSDASSLLQSLSSRLTVNTEGNRSGPQQSAPFSFSSVSGLTPLPEHYTFSIPSSPENCFGQGVSLPELQLEVSPATLDSCYIMNPFEEEWREQFELVSAHVAAMEAEEAALHGFQFFDVSSISFD |
| >DgWRKY21.1 | MQLNRGVVGGAPGCGTSDGTQLRCRERELVAQLHELLFPSPSGGSVGGASWSSTSSAQLSIEHCGSPVDAPALCGKRRGRGSKRAREGHPQEEQKQRRGSSTATKAGRGRRKKEGTATTTIVTTVPDFDGYQWKKYGQKQIEAAQYPRSYYRCTNSTDQACPAKRTVQRNDEVAADGGSPMYTVVYISEHTCKSTEAVAAPVILETTVRTKTQDIIPTVFPSSSSGISTSTQSPASSDITWSGGTVAGANATPSDRGDCSSLFVVDGDSWEWDQSPTAAAASAAALLQEMDFAGPIMSPVHVAATDGSWINELLFLNETPFVLNSCELFGF |
| >DgWRKY20.1 | MQLNRGAVGGAHGCGAPDGTQLRCRERELVAQLHELLFPSTAPSPSGRGSLGGVSWSASSSAELSVEHCSSPMKAPAPCGKQRVRGSKRVREGHPQEEQKVPAASKSATKAARGRRKKEGTATTTIVTTVPDFDGYQWKKYGQKQIEAAQYPRSYYRCTNSTDQACPAKRTVQRNDDGEGDGGSPKYTVVYISEHSCKTAEAVAAPVILETTVRTKTAADIDIAAVFPSSSSAISTGTQSPASSDITWSGGTVAGGNATTRECGDSSSLFAVDSDCLEWNPSPTASAAAAALLQEMDFAGPIMSPVHVAAADGSWINDLLFVNEAPFILNSCQLFGF |
| >DgWRKY74.1 | MCGVQPRRMEHLNDWDLQAVVRSCSGFVSSDDRAGPPPPPPPAAAETAVVKREPREAAVRGPAAKCSDASLYDLDYLDLDRKPFLLSATPPSSHQAWAAADDRHEVMISFPAAASTSGSRPRVPPGRKPGMRSTTPRPKRSKKSQLKKVVCEVPVADGGVSSDLWAWRKYGQKPIKGSPYPRGYYKCSSMKGCMARKLVERSPAKPGVLVITYMAEHCHPVPTQINALAGTTRHKSTPAEDRPTTSPKTHSRDGGDAHGEAVKCEDVSNETSAMAVDCSAEEAAAGDDDSEFWPAGLDLDEFLAPVDGDLDQAFEEDGALGRRLSL |
| >DgWRKY65.1 | MEEMADESSKYQWHDDFGFGEELMRELLDAPAMVAAADNCSSNKGADEEEEREEEGAAGARRRESMVNKLMSTVYSGPTLSDIESALSFTGVGAESQQQLFDGRKIFSPDKVLSKMENKYTMKIKTCGNGLADDGYKWRKYGQKSIKNSPNPRSYYRCTNPRCNAKKQVERAVDEPDTLVVTYEGLHLHYTYSHFFSNRPPLPPPPPPRQPRSRSCTSTPPNQQRQPRRRRPCRSTAWSSLPSPEIAAATVVVTTQASYWSTRCRNVARICTKADLLLATNGSWRTAAAVAGFWRTWCRCWSGGRHATLRQPQRRPARRGHRRRRWFPRRRPPRLLPCHGPRRPRTSTWPYSPTSSS |
| >DgWRKY0-1.1 | MCDYFLQTTEGDQHAGDLTDIVRAGGAMPVGAGNDFPSTATEWLQLPTGPILFPPAQSSSDGSGPSAADAFGDPFSGLQDPFITDYPSSSGSAAADFYDAVKNAMDIGMAKQAGFVDAAGCGAGGGAVGSVGGMLDMRNHPMFLREMPMPGVSPRAAGPYTVMGGGAPKLGVPMAAHGQAAGAPRAFDAAAGLQMSSSPRASGNVKRRKNQARKVVCIPAPAAAVPGKTTGEVVPSDLWAWRKYGQKPIKGSPYPRGYYRCSSSKGCPARKQVERCRTDSNMLVITYNSEHNHPWPTQRNVLAGSTRANYAKNSTNTASASSKNSNSSRNQHKPIVKAERKDQSAAAAAAAAVTSTATSTGSPPPMAVKEETEMDRSIGGDTSVTDDQHTDHLLQQMFSQSYRSMTPEAAGSYHHNDDFFADLTELDSDPVSLIFSTEYMETIPEKSDKEKAAANKDLHPLFMMD |
| >DgWRKY9.1 | MAMTPPTNFPPASPSSYFNNMSAGFLDSPILLTPSLFPSPTTGSFPSQPFNWMGTAPENEGLLGGVKDEQQRQYSGFTFQTTAPVPAAVAGTNAVASSFIQSSMPMAQLGQDSYNGDQQQPWSYQDAGTDGMTTRPASFSTPYEAPDMGGGNGGYSAPVSSSSGYGRVQSRRPSSDDGYNWRKYGQKQMKGSENPRSYYKCSFAGCSTKKKVEQAPDGQVTEIVYKGTHNHPKPLNPRRSSLSVPASASSYAPDASSDALSGTPENSSASYGDDETNNGVSSALAGHFGGGGQEFDDDEPDSKRWRKDGDGEGMAPVATGNRTVREPRVVVQTMSDIDILDDGYRWRKYGQKVVKGNPNPRSYYKCTTAGCPVRKHVERASQDLRAVVTTYEGKHNHDVPALRGSAAAAARYRAAAPMAPAASYHHGGGGYSSLRPDGFGAPAQAADQSGGFALSGFDYSAPSSYSYAGMQQQQQQNDAMYYDASRAKDEPRDDMFFEQSLMF |
| >DgWRKY18.1 | MSPVPSPHQSHLLGHGSRKEKRMRKVDTFAPHNDGHQWRKYGEKKINNCNFPRYYYRCTYKDNMNCPATKQIQQKDHSDPPLYQVTYYNEHSCNSAFLALTPTEFQLQTASGKAVSICFDSSGAQEPGGSPSSSAAAAAPRGAPSENKNQTTLKLRSEALSWGPGVVEQKPDLQSCSTECQDAHSANSSEDIDAGRFGSIRFFHFL |
| >DgWRKY33.1 | MSARPPPPPRPRLSLPPRSAAESLFSGTGDASPGPLTLASALFPSDADASGGGGGGGGDSGSGASSGAGPTSFTQLLIGSLSQPPPHQQTQERGRGGGVARAGPALSVAPPAGAAVFTVPPGLSPSGLFDSPGLIFSPAMGGFGMSHQQALAQVTAQASLSPLRRFDHIEQPSFSAAATSSGALQHMSSAPNMSGMSEMATTISNNDSAAFQSAEASHRYQVPAPVDKPADDGYNWRKYGQKVVKGSDCPRSYYKCTHANCPVKKKVEHAEDGQISEIIYKGKHNHQRPPNKRSKDGSSLAAEQNEQSNDTASGLSGVRRDQEAVYGMSEQLSGLSEGDDKDDKDDGESRPNEVDDRESDCKRRNIQISSQKTLTESKIIVQTTSEVDLLDDGYRWRKYGQKVVKGNPHPRSYYKCTFAGCNVRKHIERASSDPKAVITTYEGKHNHEPPVGRGNNQNGGNSASSNRSQQRGPNSMSGNQASLTRTDLSNSNQRQIGVLQFKREE |
| >DgWRKY0-2.1 | MEILEGNGGGRGNLQLLVSELCRVQELVRQLELHLQAPDTSVDMCHGLSAQIVALTDRSIGFVAAHFPDAAPSPSTTSSPLSDVSDQPFRTNTKKRKATARWTSQVRVSAAGGAEGPGDDGHSWRKYGQKDILGAKHPRGYYRCTHRNSQGCAATKQVQRADEDNALFDVVYHGQHTCRPTTGRRPPPIQHNPHAESLLYSLSAGLTVDTDHGGLHGAVSPLMPERRPVARGVSPQLMVSPAASDSYGALAMSPYPVTAYAECPSHGDLQEVVSALTTVSAPAPPPTMDAEFMPYCLFDYDLVFNVDAQPTLFL |
| >DgWRKY0-3.1 | MEILEGNGGGRGNLQLLVSELCRVQELVRQLELHLQAPDTSVDMCHGLSAQIVALTDRSIGFVAAHFPDAAPSPSTTSSPLSDVSDQPFRTNTKKRKATARWTSQVRVSAAGGAEGPGDDGHSWRKYGQKDILGAKHPRGYYRCTHRNSQGCAATKQVQRADEDNALFDVVYHGQHTCRPTTGRRPPPIQHNPHAESLLYSLSAGLTVDTDHGGLHGAVSPLMPERRPVARGVSPQLMVSPAASDSYGALAMSPYPVTAYAECPSHGDLQEVVSALTTVSAPAPPPTMDAEFMPYCLFDYDLVFNVDAQPTLFL |
| >DgWRKY16.1 | MAAHQASAGSSGDGPRRPPQRAPLSLPPRAAIDSFFASAAAAGSAAGTSPGPLTLAASLFPDMPSPAFHGSFTQLLAGAMTSPPAPSAAGPSPFAVPPGLSPAALLASPSLFSPTGNFEMSHQQALAQVTAQAVHSQYIMDSQADYSLPFSLPASALTSQHVNSSAHVTSTNETATLPSHSGNENLKSSEVSQGFQTSALAVAKPADDGYNWRKYGQKAVKGGEYPRSYYKCTQASCPVKKKVERASCGQITQIIYRGQHNHQRPPKRRSKDGGNLLNEDDFHENGDTLTRSEPGSQDHSGKVEVSNDSKRRDRGDQSSGSSDSEEENNDEAGAENGDDGVVNANKRHVPAPAQRIIVQTTSVVDLLDDGYRWRKYGQKVVKGNPHPRSYYKCTYHGCDVKKHIERCSQEPTAVITTYEGKHSHDVPAAKNSSHASAASANASSASSLPRRGQNTASSKQRSSRRAALRTSPSDSSLHLKEENEAI |
| >DgWRKY43.1 | MDVAVERLPASAPVKEEKKPETMEIRTTPPIVFESFPPTQRVDATTNKDEKLEATKAEMGEVREENERLKTLLSHIVRDYQSLQMHFHDAVKVKQQAAAAEKLPAVQPAEADVPPPMAAAADDLVSLSLGSGGYARQKGAAHERTSSSSSGTETDQEDQLSLGLSSRRSNEGGDRQASGPSAAPLLNLSSDSSADDAAPPRHTLSAAACPPASKARKSPSAGVDGADEEVLQQQAKKARVSVRVKCDTPTMNDGCQWRKYGQKISKGNPCPRAYYRCTVAPSCPVRKQVQRCADDMSILITTYEGTHSHPLPPAAAAMASTTSAAASMLLAGPSSSSHGHHLPFVSAGLLGPTTISTVASCPTVTLDLTAPHSLIQQQYTSPYAAAGYESKALPAAWSSGYLAPYGGGLPYYGKSSLPAMGQHFGMGMATTRPEQLYGAVHSSSYLQRSSSGGVHGAAAPAPAVTDTIAKAITSDPSFQSVLAAAITSYMGRGAGAAAQK |
| >DgWRKY40.1 | MEGVEEANRAAVVSCKKLVARLSQSGVDPIRLAAVAAQTDEAVSRFGKVVTILSNRVGHARARLGRRSSQPVDASCLLDYRPIPSAPYSPPANGLHLHGSSSTPSPPPPPPSRHAKEAVPVLVLTPCAGGNGKIVAPAAKPADRDRNMFLETPLLDSSGCTVPSSMATSAQIANSSKAPGAPAAATPPLCTAQFQFHQQHLHQQQQTQQHLQQQQQQRFQFEQHKPASSEKSFHIEMPAAAARSGKEPEVITFSFDNSVCTSSAATSFFTNMSSQLISMSDNSVAAPSSRKSQHCTRKADDDGCHCPKKKRPREKRVVRVPAVSDKVSDIPSDNYSWRKYGQKPIKGSPHPRGYYRCSSIKDCPARKHVERCRGDSGMLIVTYENDHNHAQPLDVSALAAHSEA |
| >DgWRKY38.1 | MPEDGYQWKKYGQKFIKNIQKIRSYFRCRDKRCAAKKKVEWHPGDPSLRIVYDGTHQHGTPSSSTGGQGEGDGEGVGNRYELSAQYFGGLPRSD |
| >DgWRKY68.1 | MEDTALASELDRLQGMARELEARVVDKNTPAAARELCGALAASVDRAVRLAGNPGKNNGQQRNSCRKATAAKVRKQVRVAAVQDTAPLDDGLSWRKYGQKDILGAPYPRAYFRCTHRHTRGCQATKQVQRAAGDPLLFDVVYHGDHTCAKAPAPSLLTSPAEQQPPVFSQEQQGSPVAAPEGIQWWSAEPVTPPGAVCPTVSSWYQHAGSYGCEAGVGLGTDMEFEAQLHEFLYPSQFFQPEIQTL |
| >DgWRKY67.1 | MDQSSSSFVAAAAGLKRRSGDVDQQERRVVVGEMDFFETEMRKEKRDRKDAASAVGHAGAADDLAINKGDLTIDMGLHVGGRRRNSGSEESTVDDGVSSNDEDYREAKAELAAAKSELARVNEENKRLKNLLNNANAKCNSLQMQITLMQQQQPQQQRSSHGHGHRDPEQQQQLELLPRQFISLGSTAHESARGSDCATVSPSSNPVPDAAMNYMGCPGKSAMVVGGAGKADLMPLPPFEHGHGVHHESGGNPETMQGWLPGKVPKFLPAAKVPEVPPPAPEAAATMRKARVSVRARSEAAMISDGCQWRKYGQKMAKGNPCPRAYYRCTMAAGCPVRKQVQRCAEDRTVLITTYEGNHNHPLPPAAMAMASTTAAAASMLLSGSTASADGLMAGGSNFLARAVLPCSSSVATISASAPFPTVTLDLTQTGASLPRQPEVAQLQAALAEAARPVSLPQLFGQTLYDQSKLSAVQAVAGTQDGGGAKIEETVNAATAKITSDPNFTAVLAAALTSYIGSSSSSGGGGAGGSSCTVQPLVSGGGDSCSREDTTAS |
| >DgWRKY2.1 | MATGQWSGIGDGGGLWAPPPALDSLFSDDSAVLGFFGGGSPAQLPSPPPLCAAALLGYPQDNFDVFHEQDLAHLAAQVAQKAELQQKLGGGLHPKIAPQLACTKYAIHDQADSSSFSLATPKLASQNVGSSVIAASMQGMTTLPSHMDSINTESTGVLQVLQGSSITLDKPADDGYNWRKYGQKAVKGGKYPRSYYKCTLNCPVRKNVEHSLDGLIIKIVYRGQHCHERPSKRFKDCGGLLNELDYLNDTEEASTRSQLDCQGYYGKTIMPIGTMGDGLLPTKEEGDEQLSGSGDSREGGDDEIGAVDGDVGDTNANERNAPGQKIIVSTTSDVDLLDDGYRWRKYGQKVVRGNPHPRSYYKCTFQGCDVKKHIERSSQEPHAVITTYEGKHIHDVPVSRNRNQATGQAYRPIPCHLLQ |
| >DgWRKY3.1 | MADPPNPSSPAGAGSPPEMPYPADRRVAALAGAGARYKAMSPARLPISREPCLTIPAGFSPSALLDSPVLLTNFKVEPSPTTGSLGMAAIMQKIAHPDIQPSPRDKSVRSSHADGGSRDFEFKPHLNSSSQSLPPATGDSKNNEHSMQNQSMNPSSSSSNMVTENRPPCSRESTLTVNVSSAPNQPVGMVGLTDSMPAKVSTSEMHQMNGSENAMQEPQSENVAEKSAEDGYNWRKYGQKHVKGSENPRSYYKCTHPDCEVKKLLERAVDGVITEVVYKGRHNHPKPQLNRRLAGGAVPSNQGEDRNDGAATADDKSSNALSIVNQVQSSGMIEPVPASVSDDDIDAGGGRPYPGDDATEEEDLDSKRRKMESAGIDAALMGKPNREPRVVVQTVSEVDILDDGYRWRKYGQKVVKGNPNPRSYYKCTSTGCPVRKHVERASHDPKSVITTYEGKHNHEVPAARNATHEMSTPPVKNTMHQINSNMPSIGGMMRACEPRNFTNHYSQAAETDTVSLDLGVGISPNHSDATKQMQSSVPDQMQYQMQSMASMYGNMRHPSMAMPTVQGNAAGRIYGSREEKANEGFTFKATPMDHSANLCYSSAGNLVMGP |
| >DgWRKY87.1 | MRDSKNSSSSSSSKLLCDSDDRAVAVLREMAKEQSQVTQLRAVVLPALQHAGGDPAEVVAQMFESILDCSQGYSRAEAPTALSFSCRRCAPAAGGGGGGNGRQEKSQEDLGWRRRQCQAQSPAAQEKVRSANYYIMRDLPMTLFCLKRLFVTTTATSGGSMGRSSSTTQITQGRLNFYRSRQSMELQNLARVRRIIRIGTSRSYYKCTYKKEQDCEATKTVQQYQENAGTDDPAMYTVVYFGQHTCKPARNDTDAAVVKTASTCLTSGGGTDELSPSDSQCSNISVTCTSVVVDHHQRTASIESNCKLDMTPPLANAEVNTSDQIFGMAAFSPLDLDTDWAIDAHGHYLL |
| >DgWRKY88.1 | MPAGTRPRSSPRCSRAYWTAAKAIAELKLLRLYRSLVDVAPLPPVAVAVAMDDKRRVRKILGGDGDNAKPNRQQRKRSRRFADDSVLLETPVRHYDGHQWRKYGQKFINNANHPRSYYKCTYKKEQDCEATKTVQQYQENAGTDDPAMYTVVYFGQHTCKPARNDTDAAVVKTASTCLTSGGGTDELSPSDSQCSNISVTCTSVVVDHHQRTASIESNCKLDMTPPLANAEVNTSDQIFGMAAFSPLDLDTDWAIDAHGHYLLKYGYW |
| >DgWRKY1.1 | MTSCGGAGDGKDGGWRSAPAVVYDDLVEAREKAAKLQAMVQGLPSGEKLMKEMVGKLSSAMSVLHTAAGVAASSSSAVGQGPGRRRKRSGAAAVSSGPHRRTSSRRRTKSPFIKMVTTTTLNDDKSWRKYGQKNIHSSSNPRSYYRCTHRPDQGCMATRQVQMSESNPSELEISYYGQHTCRDPSTFPSLIVQGAAAAGAPSDGANLISFAPINGVVASTSTSSFPHRRVKETMDHRMLFSRFSSYSSSQAQEGASHGAPSGSPSPACYEKFMQHGGGELADIPGRMMSALTVGSAPAEYWPVAGVAGVDMDAGAGMVDSFPSSPSSLGFMSGSLGSFGNNLGGDDDLFDFDS |
| >DgWRKY5.1 | MMTMDLMGRYGRAEEQVAIQEAAAAGLRGMEHLILQLSRTGTSDETSPAEHPQGQAQPQVDCREITDMTVSKFKKVISILNHRTGHARFRRGPVVAQSQGPSASEPAPAPSRAAPLASRPVPMTLDFSKTAPSAGYGNSMEYSVSGASSSFLSSVTGEGSVSNGRGGGGSSSLMLPPPPSASCGKPPLALAGGGPKRKCHDHAHSENVAGAAGGRCHCSKRRKSRVKRTTRVPAISSKAAEIPADDFSWRKYGQKPIKGSPYPRGYYKCSTVRGCPARKHVERDPNDPSMLIVTYEGDHRHTPAADQEPPLPELHKL |
| >DgWRKY4.1 | MAGTGCGDGGDWPFSADEAYADSSALLAELGWAAGFVDDGCAGELLPPLDPPPATPPGSVDGAGASSTSTDDGATLEAADADGKPAATTEAASKPTPGKTTKGPKRAWQPRFAFMTKTEIDHLEDGYRWRKYGQKAVKNSPFPRSYYRCTNSKCTVKKRVERSSDDPSVVITTYEGQHCHHTITFPRGAGAATLAGQMAFSAHHHLLYNDLPALHSPTSQNSLFCRPALSSSLLLLPQHCNRQELQAANYSTQPSSTMSLPASIPTVDKGLLDDMVPPAMRHG |
| >DgWRKY11.1 | MASIHTSNLQPNQELNQDSEPRSARATGELTPATMAVDLMGCYAPRRADDQLAIQEAAAAGLRSLELLVSSLSTKPHQPRSEQQQPFGEIADQAVSKFRKVISILDRTGHARFRRGPVEPPTAPPAPAASAHPPTPPPAAPLTVVAPVSVAAPLAVSPHPHSLTLDFTKPNLTMSAATTSVTSTSFFSSVTAGEGSVSKGRSLVSAGKPPLSGHKRKPCAGGHSEANTTGSRCHCSKRRKNRVKTTIRVPAVSAKIADIPADEYSWRKYGQKPIKGSPYPRGYYKCSTVRGCPARKHVERALDDPAMLVVTYEGEHRHSPGPSLMAPMPMAVASVSAGNGHV |
| >DgWRKY51.1 | MSSGEFQFQDELASLFTQRPGPGMQLQQQEPASGSWFADYLQAATPMPMDYDLLYRALELPVAEDSAVKKEMVLETAGGNCGAPLTPSTGGGTPNATSSMSSSSSEAGVGGGGAGDQGDSAGRLCKKEEGQGEDGKGGDDDGDKSKKGGAAAKGGKAGKGEKRPRHPRFAFMTKSEVDHLEDGYRWRKYGQKAVKNSPYPRSYYRCTTQKCVVKKRVERSFQDPAVVITTYEGKHTHPIPSALRGSTHLLAAQAAHLHHQHHHGHLGMLAQMGGGAGSLFGRSGSDVLGGLLQQQQQRARHGMAPPMIASTTQGLAGSGSIGSAGSATAAAASSHPSLQMQHFMAPDFGLLQDMLPSFIHGAGGNSNIQPSSPYGKQLH |
| >DgWRKY13.1 | MDPWIGSQPSLSLDLHVGLPPMGHHHRYQAAAPMVALAKPKLLVEETFLPPKKDPEVAVLESELQRVSEENRRLGEMLREVAAKYDALHGQFAELVASQANAAGNSNNHSHPSSASEGGSVSPSRKRKSEESLAPQEHQHHHAFSAAGAAADQAECTSGEPCKRIREECKPVVSKRYVHADPADLSLVVKDGYQWRKYGQKVTKDNPCPRAYFRCSFAPGCPVKKKVQRSAEDKTLLVATYEGEHNHSQPPPAQPQQQHDGAKNAAKPPHAPAPVSHHPQQQQPKQEAVAVVSGETAAAASELMRRNLAEQMAMTLTRDPSFKAALVSALSGRILELSPTGDVN |
| >DgWRKY69.1 | MHSRSGTGDHGGGQLYGDRQPAAGSVPVYDGDDPGSIFFQSPACGVGGDAGGGLMPPYSTITDYLQGFQDPAGLARQLDAPADQRELAVGDMIGHDGRHGRQAGGGAAAPIMTNSSMSSDLSRGRKGRPAPEEGDEEGEIDEEGSADHQNSRRDEKKQMKKKGKVEKKARGSRVAFATKSEVDHLDDGYRWRKYGQKAVKNSSFPRSYYRCTAARCGVKKQVERSQQDPSTVVTTYEGHHAHPCPAAHRGMVTTGIYSLASLQQQPHGFCPSSPDDLLLAAARAMGPPADAAAASPAALLPRQAEHRFAEYYGMQLQDVLQHGHR |
| >DgWRKY70.1 | MDSSASDWGLEAVVRSCVGGSGAVDVPSSEAAEPPPVAARQEAVVGCVAPAQPVRATASSSPLYDMLEYLDLEHEQLSRAPFSITPSSAREREVHISFPDASTSGQPLPATTRKQAGRKPSGRAHRPKRSKSKKSQVKKVVREVPVADGGISGPDDQWAWRKYGQKPIKGSPYPRGYYKCSSLKACTARKLVERSPAKPGVLIVTYIADHCHAVPTHISALAGTARNPPQSPLSDDTTKREEYSADVSSSVAADTGADGDSELWAPVEMDDMFGAFDDDFDHFFDDGDAFGRSVSL |
| >DgWRKY71.1 | MDSDDWGLGAVVRSCGGGVVPGLEAEQPSPPPVAGRREADRVQAAPACSSSPYDVLEYLDLEHERLLPRTPFSITPSSGRERDDVLISFPATLTSGPARKLVGRKPVGRAPRRPKRSKSKKSLVKKVVREVAVAEGGVSAGAGADDQWAWRKYGQKPIKGSPYPRGYYKCSSLKACPARKLVERSPAKPGVLVVTYIADHCHAVPTYINALPGTAPHSPPMSDDAASRHEDDNDSTDVSSSVAGVDDESELWAPVDMDMEPDDIFGAFNYDFDNFFDDGEDDIFRTIGFAQVCE |
| >DgWRKY22.1 | KHLLNGPVGEMDPPRAASAAGDGEDGNERVLSYGDVVLLRSDLAVLRGRHFLNDRIIAFYLAYLSSAFGADDNDDADLLLLPPSIPYLLSNLPDRDSVAMVAEPIRLASRRLALLPVNDNPDASLAEGGSHWTLLVLDAATAGASRPRFVHHDSLGVVNVPAARCLAAALRPLLPDAANGAPLVEGPTPTQVNGHDCGVYVLAVARAICNWWRNRREGGTDWFDAVRKEVTTESVKAMRAEMLDLIARLIQEKEEEKKKSTAREITVVKPVPSRPFYSFSSFSKLLNDFTTSSSTRITSPEDTATVRRPKVTRFTLPPSDLSTGIAVTTLQDGGLDATHEEILIDTEQVVSFDHMTVFDNINKPVHGVKNRLSYDGYNWRKYGQKQVKGSEFPRSYYKCTYPTCPVKRKVETTLDGQIAEIVYNGEHNHPKFHPPEKPVSPTSTKIVVTDAHDSNGTGTESQLGGRNCDFSNVAVASRSSCDCSDEFGNTSQVCDCKRSRKEKQSSMANGPTASIEAAYQSPTEREPPGDAAFRWRKYGQKAVNGNSFPRSYYRCSTARCNARKFVERSSDNSLVTTYEGRHNHGRLQ |
| >DgWRKY30.1 | MAGTSNRRGAVTEDWMLPTPSPRTLMLSLFNDDFSTDTLSDVFSDGGSDKPQDGIERSKAFADSSQGETSQATKAPLHFEPNLLVSEESSRNNGNLAEKNGFCGLKIDTSRVGYSASIRSPIMIPAGVSPRELLESPVFLPNAIAQPSPTTGKLPFLMRTSAKPTIPSVHKKAQDLSRDDRAFSFRHILRTNPPSSSIVEKVICSNDVFVLQNYSMMHWPDKICTVQSSGVTHQNETSASDSDHRIPESEPEVSEANRNGDCSSAPIIAHAEDGYNWRKYGKRQMKNSDHPTSYYKCTHHDCPVKKKVERGQDGDITEIVYKGSHNHPLPPPDSRPGGPENVPNDHFQNMHGEVGTKLSVSLYTEEPADTSEAQEIIDVSSSPSNEDVHREIHGIVSLGFDGDEDATKSKRRLDQSSDYLRKACCIPINQCLLIIIGYIEFYLRKIDSATPTTTISTIDIAALSSTAVREARVVVQTTSDVDILDDGYRWRKYGQKVVKGNPNPRSYYKCTYPGCSVRKHVERASNDLKSVITTYEGKHTHEVPAVRHPSSGPGAVPPDMQQPNNGLYRRPEPAPGSLSQFGGSSAAGFSFRMLPNGMAVPVPALGIFVPVQMAGHPPAMQNTRPVLHTGEGNVNPAARPGLPAANGNGPVAYQQLMGRLSQDHQM |
| >DgWRKY80.1 | MATSAGSLDHAAFTFTPPPFITSFTELLSGSGAGGDAAEQRSSPRGFNRGSRGGVPKFKSAQPPSLPISPPPPGTPFSYFSIPAGLSPAELLDSPVLLNYSNILASPTTGAIPAQRYDWKASADLITSQHEELSRADSGFSFHAVNSNAANSQTNSFPSFKEQQQQQVVEVSKSAAAPASNKSSGGGGNNNKQLEDGYNWRKYGQKQVKGSENPRSYYKCTYANCSMKKKVERSIADGRITQIVYKGAHDHPKPLSTRRNSSSSGCGAAAVAAAAEDHHPEHSGATPEHSSVTFGDDEADNGSQRSDGAEPEAKRWKEDADNEGSSGCGAGGKPVREPRLVVQTLSDIDILDDGFRWRKYGQKVVKGNPNPRSYYKCTTVGCPVRKHVERASHDNRAVITTYEGKHNHDVPVGRGAGASRALMPTSSETSGLIRPAATGQGPYTLEMLTNPAAGYGGAFQRTKDEPRDDLFVESLLC |
| >DgWRKY15.1 | MENYPILFATQPSSSTSSSYHFMSHGLFPVSKHEELSNSKDGSGDDTRCSPQVGGESSAGRDGGGDQADVFAVKTKREKRERRPRYAFQTRSQADILDDGYRWRKYGQKAVKDNNFPRSYYRCTHQGCNVKKQVQRLSGDEGVVVTTYEGTHTHPIEKSNENFEHILTQMQVYSTINNTSSTFTNHMFQ |
| >DgWRKY19.1 | MPYCLPNLHTVPDHYYTTPVVPLSPLQLPCHPKQLLQMQFDQDEEALMISSDHCGLYQLPALPFSNRHSSAATPSQTIVCEKTTAGFMPSFGAEEMGTSVTTRVSYEGASCNTHSGTWWRGSMAGEKGKMKVRRKMREPRFCFQTRSDVDVLDDGYKWRKYGQKVVKNSLHPRSYFRCTHSNCRVKKRVERLSTDCRMVITTYEGRHTHSPSDDTSSSGDHATACFSSF |
| >DgWRKY76.1 | MAMRRPKSEMSPPASPSPSDHRDSVIEELQKGAHLAESLRQQVELIPELGRRHAALANVSDISTALASSLSVLQSEREQYSSSSSSYAATAHAAGAFGGGGVRARNGAVARSRKAKHRRGADGQELPIKEMLTEAPENDGFHWRKYGEKKILNANFPRLYYRCGYSEEHKCPAKKYVQQQNYSDPPEFLVTLIDDHTCHTLFPAEAGRQPPSSSISANSHNCSTSQKHPSLQQLACLG |
| >DgWRKY75.1 | MAAIFTPAASSVSNLVVQGRESAAALEALLRGASFQEHGEVQELAAEILRCCDRALDALHHGGLDGAVVDDGAGGRKRKLGAAALRGPNKRARASGREAADKRVEKKWTAEDGFIWRKYGQKEIHNSKHPRLYFRCTYRDDSGCPATRQVQMSEDDPSLYVITYFGHHTCCADVAAAEASEVKDLGETHPFVINFGSAAIACSGSPKWLSAPSDDCGGRSDASRSSSQNGRLPEGGNAEEELQSKVAKVEAASSDVQPAAQPSSSGDASSSSPAWDPLATCSEWDIFNELETSFGYVSDFLQF |
| >DgWRKY42.1 | MFPSPGRAATAMAMAHAQAARTAAGGMAADSTRTAINFSFQPSDPPSSGGTLSDHHALLGFSPLVLDHPTTTSTSTIPAAPMIYRPHAPPEVLHPPRSSLSHPWSCEEDEQERQRGKGAASGRGGGGGRLHGSPSPAAAGMGVGAVRMKKSAGGGGGGAKARRKVREPRFCFKTMSDVDVLDDGYKWRKYGQKVVKNTQHPRSYYRCTQDKCRVKKRVERLAEDPRMVITTYEGRHVHSPSRDDDEDARANAEMSFIW |
