# Supplementary material for: The whole-genome and expression profile analysis of WRKY and RGAs in Dactylis glomerata showed that DG6C02319.1 and DgWRKYs may cooperate in the immunity against rust
Source: PeerJ. 2021 Aug 19;9:e11919. doi: 10.7717/peerj.11919 (PMC8380429; doi:10.7717/peerj.11919)
Supplement: Supplemental Information 3 [file peerj-09-11919-s003.docx]

**Table S3:** Transcriptome data of WRKY in orchardgrass under different tissues and stresses, including flower, leaf, root, spike, stem, heat, drought, submergence and rust stress.

**FPKM for tissues**

| id | flower | leaf | root | spike | stem |
| --- | --- | --- | --- | --- | --- |
| DgWRKY0-1.1 | 0 | 0 | 0.799595 | 0.171346 | 0.383531 |
| DgWRKY1.1 | 0 | 0.298168 | 4.412717 | 0 | 0 |
| DgWRKY2.1 | 3.588632 | 25.47004 | 14.23535 | 11.72045 | 12.09283 |
| DgWRKY3.1 | 19.20589 | 15.94751 | 20.11258 | 21.76935 | 23.2868 |
| DgWRKY4.1 | 4.673003 | 0.891985 | 2.321252 | 7.091301 | 2.420444 |
| DgWRKY5.1 | 41.70632 | 30.40214 | 188.2696 | 40.78936 | 11.2322 |
| DgWRKY6.1 | 3.597837 | 0.758661 | 1.896261 | 4.652151 | 3.840341 |
| DgWRKY7.1 | 0 | 0 | 0.663482 | 0.143758 | 0.063649 |
| DgWRKY8.1 | 3.958817 | 0.362409 | 2.583308 | 7.69155 | 6.549534 |
| DgWRKY9.1 | 0.522905 | 1.780135 | 23.74317 | 0.088015 | 0.027278 |
| DgWRKY10.1 | 6.439624 | 0 | 0.347359 | 26.82107 | 7.209812 |
| DgWRKY11.1 | 86.94104 | 26.173 | 290.8372 | 96.05966 | 26.93647 |
| DgWRKY12.1 | 0 | 0 | 1.241693 | 0 | 0 |
| DgWRKY13.1 | 12.28933 | 29.78519 | 245.4002 | 11.60176 | 6.400947 |
| DgWRKY14.1 | 2.685188 | 0.348669 | 4.475813 | 5.861339 | 2.477139 |
| DgWRKY15.1 | 2.575995 | 1.77771 | 76.55883 | 0 | 0.144717 |
| DgWRKY16.1 | 2.899062 | 2.579185 | 3.82558 | 4.499729 | 4.516837 |
| DgWRKY17.1 | 0 | 0 | 2.618202 | 0 | 0 |
| DgWRKY18.1 | 7.39537 | 1.460758 | 50.28719 | 8.634295 | 7.487816 |
| DgWRKY19.1 | 0.654768 | 0.091784 | 1.121573 | 4.898852 | 2.809399 |
| DgWRKY20.1 | 0 | 0.499652 | 2.120001 | 0 | 0.244049 |
| DgWRKY21.1 | 0 | 0 | 0.043166 | 0.026723 | 0.16564 |
| DgWRKY22.1 | 3.55449 | 0 | 0 | 0.134017 | 0 |
| DgWRKY23.1 | 0.100487 | 0.422582 | 4.78132 | 2.012761 | 0.51372 |
| DgWRKY24.1 | 1.143672 | 2.963109 | 128.2603 | 1.970165 | 1.296087 |
| DgWRKY29.1 | 7.04341 | 4.707986 | 7.528217 | 7.068148 | 2.068683 |
| DgWRKY31.1 | 0 | 0.177398 | 0.505807 | 0 | 0 |
| DgWRKY32.1 | 8.313136 | 5.015469 | 11.31108 | 10.80114 | 8.211965 |
| DgWRKY33.1 | 5.451793 | 6.127472 | 19.04004 | 4.44749 | 4.427966 |
| DgWRKY34.1 | 3.28668 | 1.698653 | 6.222762 | 5.118425 | 2.6717 |
| DgWRKY36.1 | 4.947485 | 2.977095 | 10.24314 | 2.217984 | 0.616903 |
| DgWRKY37.1 | 9.228793 | 0.581018 | 13.80529 | 20.92332 | 21.87559 |
| DgWRKY38.1 | 0.418324 | 3.674757 | 5.307855 | 0 | 0 |
| DgWRKY39.1 | 18.73901 | 12.22479 | 22.14823 | 26.09962 | 25.39653 |
| DgWRKY40.1 | 0 | 0 | 1.052767 | 0.021024 | 0.032578 |
| DgWRKY41.1 | 0.5223 | 2.489308 | 2.634292 | 0.830782 | 3.623782 |
| DgWRKY42.1 | 19.95596 | 0.848387 | 14.25857 | 24.66925 | 12.63314 |
| DgWRKY43.1 | 0 | 0 | 74.77402 | 0 | 0 |
| DgWRKY44.1 | 0.932349 | 17.02765 | 117.3537 | 0 | 1.75094 |
| DgWRKY45.1 | 20.668 | 95.94488 | 276.3546 | 5.785157 | 0.475165 |
| DgWRKY47.1 | 0.870674 | 0.521866 | 5.371347 | 0.339596 | 0 |
| DgWRKY48.1 | 0.367907 | 6.42936 | 5.648457 | 0.173393 | 0 |
| DgWRKY49.1 | 0.027125 | 0.060837 | 9.12737 | 0.20454 | 0 |
| DgWRKY50.1 | 10.92023 | 47.09276 | 20.66404 | 4.438881 | 0.135405 |
| DgWRKY51.1 | 10.57644 | 7.916365 | 42.20164 | 4.809523 | 0.687406 |
| DgWRKY53.1 | 0.052583 | 0.058967 | 19.29509 | 0 | 0 |
| DgWRKY54.1 | 47.47689 | 100.5651 | 237.2062 | 33.11374 | 20.39463 |
| DgWRKY56.1 | 0.242956 | 0.484366 | 26.51137 | 0 | 0.118292 |
| DgWRKY57.1 | 0.907614 | 2.019481 | 45.56009 | 0.80119 | 0.063129 |
| DgWRKY58.1 | 0.062749 | 0 | 12.80255 | 0.029573 | 0.091654 |
| DgWRKY59.1 | 0.068955 | 2.010505 | 4.829565 | 0.357477 | 0.050359 |
| DgWRKY60.1 | 0 | 1.269291 | 8.662219 | 0 | 0.087013 |
| DgWRKY64.1 | 1.602712 | 0.065357 | 0.443691 | 0 | 0.255383 |
| DgWRKY65.1 | 0.183954 | 0 | 0.028009 | 0.832286 | 6.529238 |
| DgWRKY66.1 | 0.215878 | 0.048418 | 12.68772 | 0.52906 | 0 |
| DgWRKY67.1 | 3.533856 | 0 | 0.18108 | 0.256229 | 0.049632 |
| DgWRKY69.1 | 2.175828 | 0.630568 | 44.70593 | 0.437836 | 0.107128 |
| DgWRKY70.1 | 0.79228 | 0.035539 | 0.14476 | 3.076799 | 0 |
| DgWRKY72.1 | 0 | 0.055118 | 0 | 0.023164 | 0 |
| DgWRKY73.1 | 3.239579 | 0 | 0.666568 | 0.247589 | 0 |
| DgWRKY74.1 | 1.698242 | 0.419624 | 8.23935 | 1.220909 | 0.168173 |
| DgWRKY77.1 | 0 | 0.280536 | 3.713735 | 0.029475 | 0.274049 |
| DgWRKY78.1 | 0.128058 | 0.179509 | 0.048746 | 0 | 0.374099 |
| DgWRKY79.1 | 6.782385 | 0 | 0.126452 | 1.487354 | 0.121307 |
| DgWRKY80.1 | 27.60361 | 95.28772 | 362.6892 | 5.846601 | 2.656336 |
| DgWRKY82.1 | 3.699799 | 19.71623 | 69.94351 | 3.822186 | 0.821429 |
| DgWRKY83.1 | 3.321984 | 10.47754 | 131.4045 | 0.065235 | 1.162525 |
| DgWRKY84.1 | 0.792269 | 0.540049 | 0.709596 | 0.444166 | 0.491635 |
| DgWRKY85.1 | 11.76071 | 6.164126 | 11.17325 | 17.29239 | 15.30362 |
| DgWRKY86.1 | 9.045696 | 13.22734 | 20.97352 | 7.101004 | 5.662566 |
| DgWRKY89.1 | 0.280685 | 0.146891 | 1.766471 | 2.804449 | 0.546645 |
| DgWRKY90.1 | 1.982307 | 7.928715 | 163.3557 | 0.249134 | 0.51475 |

**FPKM for heat stress**

| gene_name | BX_0d | BX_10d | BX_26d | 01998_0d | 01998_10d | 01998_26d |
| --- | --- | --- | --- | --- | --- | --- |
| DgWRKY3.1 | 42.06 | 115.38 | 39.8 | 133.79 | 30.75 | 39.14 |
| DgWRKY31.1 | 1.81 | 0.87 | 0.57 | 1.84 | 2.43 | 0.81 |
| DgWRKY85.1 | 13.75 | 17.48 | 12.64 | 21.41 | 21.78 | 19.84 |
| DgWRKY16.1 | 4.69 | 6.11 | 4.51 | 6.71 | 8.17 | 5.54 |
| DgWRKY6.1 | 7.22 | 12.45 | 13.31 | 20.53 | 25.84 | 5.83 |
| DgWRKY33.1 | 10.45 | 8.86 | 8.38 | 5.96 | 6.04 | 10.44 |
| DgWRKY47.1 | 1.63 | 0.53 | 0.17 | 0.98 | 0.28 | 0.71 |
| DgWRKY84.1 | 5.48 | 7.5 | 19.32 | 27.2 | 17.2 | 8.4 |
| DgWRKY32.1 | 6.64 | 4.96 | 7.48 | 4.01 | 4.55 | 3.49 |
| DgWRKY2.1 | 3.18 | 5.99 | 3.02 | 6.67 | 3.72 | 6.93 |
| DgWRKY37.1 | 3.39 | 2.06 | 2.2 | 3.16 | 4.25 | 1.56 |
| DgWRKY13.1 | 17.54 | 18.33 | 14.14 | 56.89 | 5.23 | 16.69 |
| DgWRKY51.1 | 7.38 | 9.08 | 5.53 | 12.56 | 5.29 | 6.16 |
| DgWRKY39.1 | 19.05 | 13.47 | 16.93 | 14.57 | 15.34 | 12.02 |
| DgWRKY36.1 | 2.62 | 3.95 | 0.95 | 3.63 | 1.74 | 2.73 |
| DgWRKY29.1 | 7.59 | 7.55 | 6.27 | 7.66 | 6.06 | 8.05 |
| DgWRKY54.1 | 11.68 | 9.75 | 9.95 | 16.71 | 8.66 | 11.41 |
| DgWRKY61.1 | 18.04 | 10.64 | 59.29 | 46.26 | 20.37 | 31.11 |
| DgWRKY22.1 | 0.25 | 1.63 | 0 | 1.72 | 0.34 | 5.86 |
| DgWRKY45.1 | 6.69 | 17.72 | 6.45 | 21.87 | 4.41 | 5.77 |
| DgWRKY41.1 | 2.61 | 1.99 | 1.47 | 1.57 | 4.06 | 2.41 |
| DgWRKY74.1 | 1.37 | 0.77 | 1.08 | 3.91 | 0.64 | 1.26 |
| DgWRKY34.1 | 4.12 | 5.74 | 5.28 | 6.59 | 4.03 | 5.54 |
| DgWRKY11.1 | 13.46 | 27.63 | 15.73 | 43.68 | 18.44 | 21.53 |
| DgWRKY64.1 | 4.98 | 2.53 | 1.27 | 1.12 | 1.39 | 3.4 |
| DgWRKY35.1 | 0 | 0.59 | 0 | 1.1 | 1.39 | 0.63 |
| DgWRKY80.1 | 44.05 | 94.75 | 42.55 | 141.46 | 16.46 | 55.83 |
| DgWRKY15.1 | 2.73 | 5.24 | 1.19 | 5.89 | 4.9 | 5.95 |
| DgWRKY90.1 | 3.83 | 3.84 | 2.23 | 3.55 | 0.42 | 0.75 |
| DgWRKY20.1 | 0.26 | 0.8 | 2.87 | 8.52 | 7.17 | 0.36 |
| DgWRKY8.1 | 3.88 | 3.57 | 1.98 | 1.79 | 0.67 | 2.3 |
| DgWRKY50.1 | 42.83 | 35.81 | 23.98 | 60.31 | 9.78 | 16.77 |
| DgWRKY26.1 | 1.32 | 3.39 | 0.6 | 4.16 | 0.76 | 2.3 |
| DgWRKY57.1 | 4.88 | 6.24 | 4.48 | 5.46 | 0.66 | 1.71 |
| DgWRKY86.1 | 1.71 | 2.36 | 3.85 | 4.3 | 1.61 | 3.4 |
| DgWRKY18.1 | 1.99 | 6.33 | 3.39 | 9.66 | 9.89 | 3.16 |
| DgWRKY10.1 | 0 | 0.15 | 0 | 1.07 | 0.75 | 0 |
| DgWRKY0-1.1 | 0.2 | 0.31 | 0 | 0.72 | 0.19 | 0.17 |
| DgWRKY25.1 | 39.52 | 44.99 | 26.42 | 110.72 | 16.85 | 38.29 |
| DgWRKY60.1 | 0.58 | 1.19 | 0.5 | 3.72 | 0.35 | 0.17 |
| DgWRKY4.1 | 3.78 | 3.18 | 1.79 | 3.57 | 4.5 | 5.2 |
| DgWRKY42.1 | 1.56 | 0.72 | 1.25 | 0.56 | 0.44 | 0.41 |
| DgWRKY82.1 | 9.25 | 2.51 | 6.31 | 4.29 | 0.76 | 1.83 |
| DgWRKY49.1 | 2.35 | 0.49 | 0.17 | 0 | 0 | 0.18 |
| DgWRKY68.1 | 1.15 | 1.66 | 1.07 | 0.6 | 0.31 | 0.43 |
| DgWRKY73.1 | 0.22 | 4.46 | 0 | 0 | 0.12 | 11.7 |
| DgWRKY79.1 | 0.68 | 1.03 | 0 | 0.92 | 0.96 | 0.88 |
| DgWRKY69.1 | 0.36 | 0.69 | 0 | 0.49 | 0 | 0.78 |
| DgWRKY48.1 | 17.9 | 25.7 | 21.38 | 20.79 | 10.52 | 22.8 |
| DgWRKY44.1 | 0.3 | 0.23 | 1.44 | 0.8 | 0 | 1.03 |
| DgWRKY5.1 | 16.56 | 16.62 | 23.08 | 40.85 | 9.18 | 25.09 |
| DgWRKY23.1 | 0.64 | 0.5 | 2.04 | 10.14 | 1.22 | 1.08 |
| DgWRKY14.1 | 0.58 | 0.22 | 0.69 | 2.07 | 0 | 0.74 |
| DgWRKY76.1 | 0.64 | 1.87 | 0 | 0 | 0 | 0.54 |
| DgWRKY56.1 | 0.98 | 0.63 | 0.13 | 2.2 | 0.93 | 0.99 |
| DgWRKY24.1 | 9.34 | 13.42 | 3.22 | 8.42 | 1.08 | 7.89 |
| DgWRKY19.1 | 3.54 | 0.84 | 0.93 | 1.05 | 1.08 | 0.97 |
| DgWRKY70.1 | 1.2 | 0 | 0 | 6.41 | 0 | 0 |
| DgWRKY89.1 | 2.09 | 0.74 | 0 | 0.93 | 0 | 0 |
| DgWRKY72.1 | 0.21 | 1.58 | 1.48 | 1.45 | 0 | 1.6 |

**TPM for drought stress**

| gene_name | L_18d_CK | L_18d_D | R_18d_CK | R_18d_D |
| --- | --- | --- | --- | --- |
| DgWRKY3.1 | 28.305 | 21.935 | 20.985 | 27.21 |
| DgWRKY85.1 | 12.32 | 11.77 | 11.105 | 17.555 |
| DgWRKY43.1 | 0.785 | 0 | 115.71 | 15.435 |
| DgWRKY80.1 | 453.935 | 431.59 | 557.34 | 849.23 |
| DgWRKY45.1 | 80.895 | 133.48 | 194.745 | 129.82 |
| DgWRKY2.1 | 6.445 | 6.21 | 10.07 | 4.87 |
| DgWRKY6.1 | 6.425 | 16.84 | 54.575 | 34.05 |
| DgWRKY37.1 | 5.62 | 3.48 | 36.795 | 1.35 |
| DgWRKY13.1 | 132.245 | 169.585 | 900.73 | 621.855 |
| DgWRKY29.1 | 26.275 | 19.14 | 70.295 | 43.61 |
| DgWRKY14.1 | 1.085 | 0.37 | 63.65 | 36.615 |
| DgWRKY82.1 | 39.995 | 4.665 | 21.325 | 17.595 |
| DgWRKY90.1 | 91.065 | 56.515 | 375.935 | 165.375 |
| DgWRKY39.1 | 34.26 | 31.02 | 52.26 | 230.075 |
| DgWRKY36.1 | 16.605 | 16.885 | 87.03 | 30.06 |
| DgWRKY54.1 | 20.14 | 35.825 | 44.645 | 108.545 |
| DgWRKY60.1 | 0.68 | 0.48 | 57.415 | 17.34 |
| DgWRKY47.1 | 3.135 | 0.945 | 10.55 | 1.365 |
| DgWRKY31.1 | 13.125 | 2.91 | 7.29 | 4.85 |
| DgWRKY1.1 | 7.805 | 0.47 | 18.975 | 5.79 |
| DgWRKY64.1 | 8.055 | 16.79 | 0.58 | 4.435 |
| DgWRKY49.1 | 24.56 | 36.755 | 95.655 | 583.3 |
| DgWRKY16.1 | 5.425 | 5.115 | 23.285 | 16.2 |
| DgWRKY11.1 | 220.025 | 95.98 | 208.675 | 564.515 |
| DgWRKY34.1 | 9.53 | 9.87 | 16.81 | 89.385 |
| DgWRKY5.1 | 66.575 | 25.32 | 156.2 | 240.5 |
| DgWRKY18.1 | 5.64 | 21.47 | 66.78 | 301.12 |
| DgWRKY57.1 | 96.255 | 19.175 | 164.88 | 161.075 |
| DgWRKY83.1 | 63.395 | 9.295 | 59.28 | 28.95 |
| DgWRKY26.1 | 11.695 | 3.675 | 19.17 | 123.875 |
| DgWRKY15.1 | 41.9 | 29.57 | 61.89 | 224.315 |
| DgWRKY50.1 | 27.665 | 5.12 | 34.88 | 63.565 |
| DgWRKY17.1 | 0.865 | 0.135 | 3.84 | 6.67 |
| DgWRKY48.1 | 22.01 | 12.485 | 3.245 | 0 |
| DgWRKY75.1 | 0.335 | 0.345 | 21.09 | 6.88 |
| DgWRKY22.1 | 8.73 | 6.18 | 4.87 | 6 |
| DgWRKY88.1 | 0 | 0 | 91.47 | 16.265 |
| DgWRKY81.1 | 1.14 | 0.18 | 20.02 | 1.505 |
| DgWRKY32.1 | 7.89 | 5.295 | 8.37 | 4.215 |
| DgWRKY24.1 | 293.49 | 100.47 | 969.57 | 279.26 |
| DgWRKY12.1 | 0 | 0 | 6.525 | 8.575 |
| DgWRKY56.1 | 4.7 | 2.08 | 13.28 | 5.2 |
| DgWRKY70.1 | 3 | 0.435 | 6.585 | 8.11 |
| DgWRKY59.1 | 0 | 0.185 | 24.175 | 1.94 |
| DgWRKY44.1 | 24.24 | 1.365 | 0.565 | 0.135 |
| DgWRKY42.1 | 4.265 | 12.36 | 11.075 | 11.725 |
| DgWRKY86.1 | 1.645 | 0.655 | 8.005 | 13.96 |
| DgWRKY41.1 | 5.14 | 2.9 | 0.35 | 0.09 |
| DgWRKY89.1 | 1.2 | 0 | 4.62 | 19.725 |
| DgWRKY7.1 | 0 | 0 | 3.92 | 7.43 |
| DgWRKY72.1 | 44.585 | 5.75 | 11.665 | 10.49 |
| DgWRKY68.1 | 16.935 | 14.375 | 49.46 | 6.69 |
| DgWRKY23.1 | 36.985 | 16.235 | 75.355 | 3.61 |
| DgWRKY8.1 | 9.865 | 8.68 | 10.325 | 0.27 |
| DgWRKY38.1 | 4.9 | 4.655 | 4.265 | 6.67 |
| DgWRKY84.1 | 7.385 | 2.025 | 3.15 | 5.24 |
| DgWRKY9.1 | 38.9 | 5.685 | 40.155 | 51.18 |
| DgWRKY0-1.1 | 1.695 | 0 | 5.02 | 3.555 |
| DgWRKY66.1 | 0.23 | 0.2 | 0.095 | 10.64 |
| DgWRKY4.1 | 5.945 | 2.125 | 7.335 | 2.92 |
| DgWRKY40.1 | 0.095 | 0 | 7.325 | 5.785 |
| DgWRKY78.1 | 0 | 0 | 4.495 | 8.5 |
| DgWRKY67.1 | 0 | 3.99 | 0 | 0 |

**FPKM for submergence stress**

| gene name | Dianbei_8h_CK | Dianbei_8h_S | Dianbei_24h_CK | Dianbei_24h_S | Anba_8h_CK | Anba_8h_S | Anba_24h_CK | Anba_24h_S |
| --- | --- | --- | --- | --- | --- | --- | --- | --- |
| DgWRKY84.1 | 0.017 | 0.285 | 0.204 | 0.059 | 3.096 | 1.327 | 2.079 | 1.965 |
| DgWRKY6.1 | 1.157 | 2.011 | 1.564 | 2.582 | 1.641 | 2.099 | 0.437 | 1.009 |
| DgWRKY10.1 | 0.013 | 0.004 | 0.004 | 0.059 | 0.000 | 0.019 | 0.000 | 0.000 |
| DgWRKY29.1 | 17.571 | 23.361 | 14.408 | 24.892 | 15.493 | 19.481 | 16.316 | 18.499 |
| DgWRKY3.1 | 34.634 | 52.951 | 30.920 | 72.192 | 23.221 | 38.292 | 24.555 | 39.017 |
| DgWRKY31.1 | 1.446 | 4.439 | 0.576 | 4.314 | 0.224 | 0.802 | 1.548 | 1.243 |
| DgWRKY12.1 | 0.000 | 0.341 | 0.006 | 0.000 | 0.000 | 0.000 | 0.012 | 0.000 |
| DgWRKY67.1 | 0.071 | 0.069 | 0.012 | 0.119 | 0.151 | 0.082 | 0.012 | 0.016 |
| DgWRKY57.1 | 9.593 | 41.991 | 12.561 | 44.873 | 2.675 | 85.975 | 6.953 | 13.984 |
| DgWRKY47.1 | 5.257 | 14.530 | 2.247 | 6.494 | 4.796 | 4.960 | 5.410 | 5.035 |
| DgWRKY85.1 | 19.340 | 21.549 | 14.874 | 21.530 | 20.730 | 20.506 | 16.898 | 18.948 |
| DgWRKY33.1 | 12.070 | 24.382 | 9.578 | 19.647 | 15.352 | 16.972 | 12.267 | 16.720 |
| DgWRKY9.1 | 13.693 | 13.633 | 3.905 | 15.783 | 4.417 | 77.675 | 4.532 | 5.702 |
| DgWRKY43.1 | 1.317 | 16.607 | 0.194 | 14.506 | 0.224 | 3.234 | 0.101 | 0.865 |
| DgWRKY16.1 | 6.447 | 7.667 | 4.576 | 6.114 | 9.533 | 6.687 | 6.031 | 5.836 |
| DgWRKY80.1 | 249.640 | 365.801 | 64.931 | 312.131 | 60.685 | 591.111 | 32.428 | 142.277 |
| DgWRKY0-1.1 | 0.133 | 0.315 | 0.104 | 1.155 | 0.198 | 0.176 | 0.746 | 0.367 |
| DgWRKY45.1 | 98.018 | 155.330 | 12.979 | 60.982 | 63.625 | 176.321 | 22.526 | 51.328 |
| DgWRKY2.1 | 20.314 | 19.344 | 2.537 | 9.588 | 22.487 | 17.604 | 1.652 | 5.438 |
| DgWRKY35.1 | 0.010 | 0.018 | 0.139 | 0.050 | 0.037 | 0.067 | 0.618 | 0.257 |
| DgWRKY37.1 | 1.101 | 1.725 | 0.983 | 2.985 | 1.189 | 1.253 | 1.437 | 2.022 |
| DgWRKY51.1 | 25.789 | 50.941 | 22.885 | 64.136 | 20.395 | 35.280 | 28.503 | 38.966 |
| DgWRKY89.1 | 0.401 | 0.261 | 0.194 | 0.047 | 0.343 | 0.213 | 0.209 | 0.119 |
| DgWRKY32.1 | 8.838 | 13.656 | 9.549 | 11.707 | 8.329 | 9.274 | 5.861 | 9.997 |
| DgWRKY65.1 | 0.019 | 0.082 | 0.007 | 0.150 | 0.036 | 0.061 | 0.007 | 0.034 |
| DgWRKY81.1 | 0.150 | 2.176 | 0.124 | 1.141 | 0.081 | 0.748 | 0.212 | 0.499 |
| DgWRKY1.1 | 0.231 | 54.462 | 0.427 | 22.702 | 0.077 | 9.631 | 0.392 | 5.360 |
| DgWRKY49.1 | 0.303 | 1.501 | 11.720 | 0.255 | 0.181 | 0.059 | 0.270 | 0.505 |
| DgWRKY13.1 | 47.701 | 58.835 | 32.526 | 128.048 | 18.680 | 359.922 | 27.229 | 122.445 |
| DgWRKY11.1 | 50.372 | 71.575 | 25.062 | 123.282 | 21.512 | 147.059 | 27.636 | 72.016 |
| DgWRKY50.1 | 44.737 | 59.940 | 23.558 | 38.445 | 28.068 | 129.796 | 20.740 | 16.935 |
| DgWRKY79.1 | 0.000 | 0.022 | 0.051 | 0.000 | 0.029 | 0.010 | 0.000 | 0.000 |
| DgWRKY24.1 | 25.430 | 13.418 | 5.279 | 79.762 | 12.662 | 126.716 | 7.117 | 37.878 |
| DgWRKY90.1 | 44.206 | 65.033 | 19.061 | 122.287 | 0.846 | 189.396 | 3.541 | 31.056 |
| DgWRKY20.1 | 0.151 | 0.060 | 0.556 | 0.224 | 0.000 | 2.867 | 0.093 | 2.372 |
| DgWRKY39.1 | 19.235 | 41.163 | 15.391 | 32.953 | 21.422 | 40.922 | 18.639 | 31.252 |
| DgWRKY14.1 | 1.724 | 2.220 | 1.290 | 1.567 | 1.987 | 2.142 | 1.636 | 1.227 |
| DgWRKY21.1 | 0.036 | 0.226 | 0.496 | 0.174 | 0.009 | 0.776 | 0.088 | 1.597 |
| DgWRKY74.1 | 1.364 | 3.028 | 0.359 | 2.061 | 1.481 | 2.767 | 1.232 | 1.032 |
| DgWRKY69.1 | 0.196 | 1.830 | 0.325 | 1.004 | 0.232 | 0.350 | 0.186 | 0.393 |
| DgWRKY22.1 | 0.171 | 0.148 | 0.181 | 0.073 | 0.095 | 0.407 | 0.521 | 0.315 |
| DgWRKY64.1 | 2.173 | 1.679 | 3.921 | 3.625 | 2.347 | 2.423 | 3.836 | 1.937 |
| DgWRKY26.1 | 0.070 | 3.079 | 1.103 | 1.299 | 0.032 | 2.521 | 0.604 | 2.940 |
| DgWRKY82.1 | 10.930 | 70.131 | 18.542 | 99.576 | 24.849 | 103.718 | 19.431 | 55.608 |
| DgWRKY60.1 | 0.173 | 0.332 | 0.346 | 0.467 | 0.080 | 0.239 | 0.382 | 0.097 |
| DgWRKY5.1 | 27.923 | 79.615 | 28.297 | 79.818 | 28.587 | 119.426 | 34.965 | 74.853 |
| DgWRKY17.1 | 0.000 | 6.431 | 0.302 | 2.835 | 0.021 | 10.603 | 0.122 | 0.452 |
| DgWRKY36.1 | 6.930 | 17.469 | 7.262 | 13.644 | 4.883 | 11.522 | 6.146 | 7.004 |
| DgWRKY40.1 | 0.000 | 0.000 | 0.000 | 0.021 | 0.008 | 0.033 | 0.009 | 0.046 |
| DgWRKY54.1 | 88.142 | 166.919 | 12.080 | 96.935 | 45.515 | 120.287 | 18.657 | 68.155 |
| DgWRKY23.1 | 3.281 | 5.090 | 3.132 | 4.917 | 0.151 | 5.591 | 0.174 | 1.742 |
| DgWRKY77.1 | 0.110 | 3.889 | 0.102 | 1.013 | 0.065 | 3.307 | 0.000 | 1.019 |
| DgWRKY58.1 | 0.011 | 0.762 | 0.045 | 0.105 | 0.041 | 0.813 | 0.059 | 0.148 |
| DgWRKY86.1 | 14.478 | 21.071 | 7.154 | 14.588 | 16.235 | 24.124 | 11.245 | 17.361 |
| DgWRKY25.1 | 54.313 | 24.443 | 16.492 | 65.887 | 4.614 | 62.288 | 3.291 | 13.456 |
| DgWRKY70.1 | 0.000 | 0.040 | 0.000 | 0.038 | 0.023 | 0.046 | 0.025 | 0.061 |
| DgWRKY78.1 | 0.013 | 0.741 | 0.300 | 0.395 | 0.000 | 0.758 | 0.159 | 0.081 |
| DgWRKY56.1 | 6.849 | 13.171 | 3.062 | 10.786 | 3.006 | 8.544 | 6.608 | 11.357 |
| DgWRKY4.1 | 5.968 | 2.834 | 4.479 | 4.455 | 7.356 | 2.154 | 8.132 | 2.991 |
| DgWRKY59.1 | 0.221 | 0.701 | 0.136 | 0.285 | 0.000 | 0.292 | 0.000 | 0.012 |
| DgWRKY28.1 | 0.000 | 0.532 | 0.000 | 0.057 | 0.000 | 0.128 | 0.000 | 0.000 |
| DgWRKY72.1 | 0.000 | 0.404 | 0.000 | 0.000 | 0.000 | 0.854 | 0.000 | 0.562 |
| DgWRKY61.1 | 0.000 | 0.030 | 0.302 | 0.000 | 0.044 | 0.000 | 0.000 | 0.000 |
| DgWRKY42.1 | 1.997 | 2.991 | 3.254 | 2.236 | 1.118 | 1.588 | 2.648 | 1.802 |
| DgWRKY68.1 | 33.565 | 16.062 | 28.082 | 20.246 | 25.359 | 27.017 | 25.173 | 18.113 |
| DgWRKY76.1 | 0.017 | 0.018 | 0.088 | 0.016 | 0.000 | 0.000 | 0.015 | 0.016 |
| DgWRKY48.1 | 8.463 | 12.710 | 5.879 | 8.337 | 8.491 | 11.962 | 3.277 | 3.177 |
| DgWRKY8.1 | 2.374 | 2.183 | 2.878 | 2.313 | 2.802 | 2.887 | 2.347 | 3.808 |
| DgWRKY19.1 | 0.650 | 2.203 | 0.811 | 0.252 | 1.066 | 1.369 | 0.519 | 0.608 |
| DgWRKY30.1 | 0.117 | 0.036 | 0.012 | 0.000 | 0.071 | 0.063 | 0.267 | 0.017 |
| DgWRKY34.1 | 4.404 | 5.058 | 4.715 | 3.319 | 3.959 | 3.391 | 2.891 | 1.640 |
| DgWRKY66.1 | 0.500 | 2.162 | 0.966 | 1.007 | 0.238 | 0.316 | 0.265 | 0.560 |
| DgWRKY73.1 | 0.127 | 0.000 | 0.030 | 0.000 | 0.014 | 0.000 | 0.082 | 0.000 |
| DgWRKY44.1 | 13.829 | 18.486 | 7.184 | 49.670 | 2.008 | 45.765 | 7.328 | 44.103 |
| DgWRKY41.1 | 4.189 | 3.455 | 3.663 | 6.000 | 5.296 | 4.977 | 4.099 | 4.219 |
| DgWRKY18.1 | 6.729 | 22.738 | 5.683 | 27.570 | 11.562 | 16.582 | 16.319 | 26.532 |
| DgWRKY83.1 | 15.669 | 24.419 | 28.580 | 107.152 | 5.554 | 58.509 | 20.584 | 58.905 |
| DgWRKY15.1 | 30.035 | 157.593 | 14.926 | 116.820 | 11.431 | 83.623 | 13.843 | 64.823 |
| DgWRKY38.1 | 0.435 | 10.047 | 0.793 | 3.103 | 1.348 | 3.015 | 0.720 | 1.476 |

**FPKM for rust stress**

| gene_name | HS_7d_CK | HS_7d_R | HS_14d_CK | HS_14d_R | HR_7d_CK | HR_7d_R | HR_14d_CK | HR_14d_R |
| --- | --- | --- | --- | --- | --- | --- | --- | --- |
| DgWRKY0-1.1 | 0.030 | 0.440 | 0.000 | 0.000 | 0.000 | 0.795 | 0.000 | 0.585 |
| DgWRKY1.1 | 1.397 | 5.467 | 15.393 | 0.535 | 0.033 | 90.735 | 6.350 | 57.630 |
| DgWRKY2.1 | 10.873 | 6.487 | 7.813 | 10.675 | 5.067 | 14.880 | 5.580 | 19.745 |
| DgWRKY3.1 | 38.883 | 66.923 | 45.180 | 29.320 | 20.903 | 43.440 | 32.575 | 55.400 |
| DgWRKY4.1 | 11.787 | 9.467 | 12.367 | 16.685 | 5.667 | 11.460 | 1.115 | 3.030 |
| DgWRKY5.1 | 31.520 | 154.220 | 265.103 | 22.210 | 49.483 | 572.565 | 51.155 | 161.360 |
| DgWRKY6.1 | 0.813 | 1.200 | 0.910 | 0.235 | 0.457 | 2.145 | 1.810 | 2.790 |
| DgWRKY8.1 | 2.877 | 4.893 | 19.210 | 3.040 | 2.113 | 1.470 | 1.005 | 5.605 |
| DgWRKY9.1 | 0.887 | 14.350 | 18.800 | 1.100 | 1.567 | 86.885 | 1.045 | 29.450 |
| DgWRKY10.1 | 0.097 | 0.103 | 0.070 | 0.000 | 0.050 | 0.110 | 0.000 | 0.000 |
| DgWRKY11.1 | 28.250 | 77.443 | 114.060 | 41.220 | 49.177 | 449.955 | 34.045 | 128.600 |
| DgWRKY12.1 | 0.000 | 0.077 | 0.083 | 0.000 | 0.000 | 0.000 | 0.425 | 3.715 |
| DgWRKY13.1 | 8.407 | 73.963 | 111.680 | 7.575 | 101.373 | 1296.265 | 141.990 | 315.235 |
| DgWRKY14.1 | 1.033 | 2.887 | 2.627 | 0.210 | 0.700 | 22.125 | 1.635 | 3.170 |
| DgWRKY15.1 | 3.747 | 59.473 | 155.867 | 2.340 | 1.650 | 68.725 | 16.565 | 282.820 |
| DgWRKY16.1 | 4.143 | 7.133 | 4.733 | 3.240 | 7.297 | 5.510 | 3.600 | 4.650 |
| DgWRKY17.1 | 0.177 | 1.930 | 19.347 | 2.190 | 1.107 | 20.740 | 11.020 | 64.530 |
| DgWRKY18.1 | 5.393 | 72.107 | 140.927 | 3.125 | 2.680 | 29.415 | 16.855 | 144.885 |
| DgWRKY19.1 | 0.127 | 0.603 | 0.753 | 0.395 | 0.870 | 0.175 | 1.255 | 0.975 |
| DgWRKY20.1 | 1.037 | 0.967 | 12.737 | 3.490 | 92.493 | 56.495 | 1.805 | 7.795 |
| DgWRKY21.1 | 0.307 | 0.220 | 13.873 | 0.950 | 43.717 | 22.840 | 0.000 | 7.190 |
| DgWRKY22.1 | 0.043 | 0.337 | 0.000 | 0.000 | 0.000 | 0.050 | 0.000 | 0.575 |
| DgWRKY23.1 | 1.493 | 4.357 | 9.113 | 4.050 | 1.033 | 26.055 | 0.200 | 1.435 |
| DgWRKY24.1 | 11.853 | 16.253 | 95.920 | 44.320 | 36.417 | 586.350 | 58.545 | 72.465 |
| DgWRKY28.1 | 0.643 | 0.467 | 5.907 | 0.350 | 0.000 | 4.395 | 1.355 | 17.060 |
| DgWRKY29.1 | 10.810 | 18.390 | 9.220 | 6.305 | 4.240 | 13.575 | 13.680 | 16.045 |
| DgWRKY30.1 | 0.000 | 0.000 | 0.200 | 0.000 | 0.130 | 0.255 | 0.000 | 0.130 |
| DgWRKY31.1 | 1.377 | 6.583 | 5.610 | 1.015 | 0.033 | 4.985 | 0.000 | 8.970 |
| DgWRKY32.1 | 8.820 | 14.050 | 12.713 | 6.090 | 5.637 | 6.500 | 7.920 | 10.875 |
| DgWRKY33.1 | 8.397 | 20.553 | 25.793 | 4.825 | 7.610 | 19.290 | 21.675 | 38.080 |
| DgWRKY34.1 | 3.437 | 5.183 | 9.070 | 2.965 | 2.700 | 9.775 | 4.345 | 8.375 |
| DgWRKY35.1 | 0.460 | 0.787 | 0.180 | 0.845 | 0.000 | 0.130 | 0.000 | 0.000 |
| DgWRKY36.1 | 23.957 | 26.813 | 16.683 | 12.360 | 11.127 | 114.470 | 8.195 | 11.875 |
| DgWRKY37.1 | 5.860 | 16.567 | 4.337 | 2.605 | 1.303 | 5.735 | 0.345 | 2.935 |
| DgWRKY38.1 | 5.950 | 3.420 | 7.027 | 6.975 | 3.703 | 41.810 | 9.235 | 89.045 |
| DgWRKY39.1 | 24.047 | 50.003 | 58.933 | 19.245 | 12.217 | 51.040 | 31.505 | 58.825 |
| DgWRKY40.1 | 0.000 | 0.140 | 0.000 | 0.000 | 0.000 | 0.150 | 0.000 | 0.000 |
| DgWRKY41.1 | 2.057 | 8.573 | 9.233 | 1.410 | 8.930 | 1.610 | 5.040 | 4.875 |
| DgWRKY42.1 | 0.903 | 2.670 | 4.780 | 1.025 | 1.417 | 0.970 | 0.425 | 1.315 |
| DgWRKY43.1 | 0.000 | 9.470 | 20.723 | 0.000 | 0.020 | 42.630 | 2.745 | 63.545 |
| DgWRKY44.1 | 4.957 | 12.033 | 17.307 | 10.085 | 6.007 | 21.395 | 14.605 | 66.140 |
| DgWRKY45.1 | 23.307 | 82.657 | 109.467 | 21.630 | 4.760 | 159.875 | 30.790 | 112.755 |
| DgWRKY47.1 | 1.170 | 8.907 | 21.890 | 0.870 | 2.833 | 25.405 | 4.770 | 58.535 |
| DgWRKY48.1 | 8.550 | 10.500 | 21.370 | 3.765 | 8.147 | 36.970 | 8.535 | 56.635 |
| DgWRKY49.1 | 0.620 | 20.997 | 13.643 | 0.085 | 0.067 | 10.540 | 0.670 | 4.915 |
| DgWRKY50.1 | 20.380 | 22.757 | 35.713 | 14.215 | 16.320 | 247.755 | 19.790 | 73.655 |
| DgWRKY51.1 | 9.453 | 66.757 | 36.023 | 5.680 | 10.227 | 40.415 | 5.365 | 43.755 |
| DgWRKY53.1 | 0.000 | 0.000 | 0.000 | 0.000 | 0.390 | 1.550 | 0.950 | 0.000 |
| DgWRKY54.1 | 45.213 | 83.493 | 107.373 | 45.240 | 13.473 | 32.325 | 121.120 | 239.160 |
| DgWRKY56.1 | 0.713 | 10.667 | 11.363 | 0.580 | 0.593 | 25.315 | 5.240 | 20.215 |
| DgWRKY57.1 | 2.587 | 29.650 | 54.167 | 3.215 | 9.943 | 297.060 | 9.940 | 93.980 |
| DgWRKY58.1 | 0.187 | 0.457 | 3.213 | 0.105 | 0.670 | 3.230 | 0.000 | 0.075 |
| DgWRKY59.1 | 0.657 | 0.543 | 0.320 | 0.000 | 0.567 | 6.790 | 0.230 | 3.075 |
| DgWRKY60.1 | 0.177 | 0.000 | 1.190 | 0.225 | 2.063 | 12.060 | 0.260 | 1.610 |
| DgWRKY62.1 | 0.000 | 0.000 | 0.000 | 0.000 | 0.000 | 0.000 | 0.000 | 0.345 |
| DgWRKY63.1 | 0.000 | 0.000 | 0.000 | 0.000 | 0.000 | 0.000 | 0.000 | 0.270 |
| DgWRKY64.1 | 0.933 | 2.843 | 1.653 | 0.315 | 0.593 | 0.525 | 0.810 | 0.810 |
| DgWRKY65.1 | 0.010 | 0.653 | 0.680 | 0.080 | 0.000 | 0.035 | 0.070 | 0.360 |
| DgWRKY66.1 | 0.000 | 4.740 | 7.827 | 0.000 | 0.467 | 7.935 | 0.000 | 13.025 |
| DgWRKY67.1 | 0.063 | 0.050 | 0.373 | 0.140 | 0.000 | 0.565 | 0.245 | 0.360 |
| DgWRKY69.1 | 0.787 | 17.777 | 26.213 | 1.030 | 0.940 | 12.875 | 2.180 | 20.415 |
| DgWRKY70.1 | 0.000 | 0.000 | 0.000 | 0.000 | 0.000 | 0.405 | 0.000 | 0.445 |
| DgWRKY72.1 | 0.500 | 0.287 | 2.683 | 0.270 | 0.550 | 1.560 | 0.500 | 2.470 |
| DgWRKY73.1 | 0.030 | 0.280 | 0.000 | 0.000 | 0.473 | 0.000 | 0.000 | 0.000 |
| DgWRKY74.1 | 1.843 | 3.813 | 6.413 | 1.425 | 4.180 | 51.925 | 8.930 | 11.860 |
| DgWRKY76.1 | 0.283 | 0.623 | 0.260 | 1.040 | 0.703 | 4.015 | 0.380 | 8.430 |
| DgWRKY77.1 | 1.007 | 2.343 | 36.040 | 0.680 | 17.020 | 61.955 | 8.265 | 98.735 |
| DgWRKY78.1 | 1.640 | 2.477 | 22.867 | 2.730 | 3.393 | 17.050 | 1.640 | 41.945 |
| DgWRKY79.1 | 0.113 | 0.467 | 0.000 | 0.000 | 0.000 | 0.195 | 0.000 | 0.245 |
| DgWRKY80.1 | 68.510 | 188.720 | 414.793 | 99.150 | 54.840 | 381.975 | 65.265 | 256.545 |
| DgWRKY81.1 | 0.000 | 1.013 | 0.863 | 0.000 | 0.000 | 10.980 | 0.000 | 3.660 |
| DgWRKY82.1 | 31.853 | 31.653 | 255.137 | 30.565 | 82.273 | 447.800 | 202.690 | 963.235 |
| DgWRKY83.1 | 24.163 | 48.460 | 208.797 | 63.350 | 32.190 | 179.760 | 6.265 | 210.245 |
| DgWRKY84.1 | 1.353 | 2.040 | 0.950 | 0.875 | 1.933 | 1.840 | 2.255 | 2.345 |
| DgWRKY85.1 | 14.280 | 26.090 | 27.560 | 12.015 | 13.143 | 20.790 | 34.470 | 31.395 |
| DgWRKY86.1 | 6.373 | 11.577 | 8.860 | 5.860 | 4.263 | 20.005 | 12.115 | 16.675 |
| DgWRKY89.1 | 0.260 | 0.487 | 2.980 | 0.000 | 0.297 | 0.060 | 0.000 | 0.000 |
| DgWRKY90.1 | 5.340 | 43.907 | 121.497 | 8.720 | 71.230 | 764.455 | 18.305 | 153.955 |
