# Supplementary material for: The whole-genome and expression profile analysis of WRKY and RGAs in Dactylis glomerata showed that DG6C02319.1 and DgWRKYs may cooperate in the immunity against rust
Source: PeerJ. 2021 Aug 19;9:e11919. doi: 10.7717/peerj.11919 (PMC8380429; doi:10.7717/peerj.11919)
Supplement: Supplemental Information 4 [file peerj-09-11919-s004.docx]

**Table S4:** The promoter sequence of the RGAs.

>DG2C02110.1 - Up_Stream_Len 1700

ctctgcagaatagcttttcactgatggattactcgatggagatgctttccttgggatgta

actgttgctgtgattccaattatgttgcctgtaatttgtcctgatcagtttgtgtctttg

gattagccggcagctggaggaggaagaaactagagggggaagggaggagtggaggaaaga

gagcagatggagtttggacgggaggggtttggtagggtttcttccctgggatcacgtgca

tggggcgcgcgggggtggtcgtggccgcgcgcaggggcgcacttggcgtttagccagaac

aggaaattttgcgggaaggcagaactttccattttcaaaaagtttgggatccgacccggg

ggcaccgcgtaagacattttggtaaccgcgcactgtgtatccgcgtcctatcattaaacc

ctatgcacatggataaataagggaaagcagcatccaaaaatagcaatgagagacagtgca

gttacacctaatacagagcatccgcacatcatcatccaaaaactaaaactgaacctgtac

ctagaaagaaaactgaatttttagcactaattattcagctaagttattccggtatagtga

atacatggttccgttccgtttcaaagaccatgtccacacagcacaagcgttcccacaagt

accagagccaagtgatcaaccagcctgctgccactgccacaactcacacgctacccagta

tactttggcaagcattccaatccagctgatacagcgcagcaagcatcagcacatcaccca

aaaactagaatacacctgcaccgaaaacaccaattagaatcccccacaaacatagagacg

agcgatcgaccagccaactactgccagaactcacacgctacccagtttactttgacaaac

attcaaattcagatggtccacaatagtgcgccagtcgaactaggagaaaatagaaaagag

cagaaaacgtatgcatcggacaagtcgaagtaaacgcctcaggccaaggagatggaagag

agcacagatcacggccattaggcaaaaaaggattgcactttcttcttgccctccacttgg

gtttgtccatgcactcactgagatggctatgagtgctcccgatggcaagcccctttcagt

gatggtcttcccgtgaactatctcgccggaagaattttcagcaaatcttcagcagctgtt

tccatgtcaaatcgtgcctttccctgttcaccaaagggcaaaggatgcattatgaaaata

agaagtaatatttttggtcggtgctagagcgaaaatgttacatttgtttagtgaagctgg

taagagaagtgatgtcaggtcaagcaaattaaggcacgctgtaagacttcagcaaggacc

aagtcaaggataaaactgtaaagtctatagagggcttatctggatggtatacgatcggta

ttcgagaagatgtgggcatttgacttgtcactagattacagcaggcagccaatagtgaag

cgctgaacatcatcagtgtgaaagaggaagctacctggtgggctgagggcagttgtgtaa

aatcccattctcttgacccagaattctgtgctgtgactacgactacaagatgtgctcctt

catgattagaccaccaatgatgctttatccactcagtgtacaacataagctctggcccat

gctagttccaccagaaaacc

>DG3C03908.1 + Up_Stream_Len 1700

cttgttgtctagtaaaaagacaactgattttctgtcagacttttttgttataaaattttg

taaaaaaaatgtttggattttcagaaaaatatgattttgaaaaatcataataaattttaa

aaaatatttggtgaacacaaaaaagtttggatttgaaaaaaaagcctacatgagattttc

agattctaaaattgatttcaaaataaaacgttagaaaaattatccaaattataaaatatt

catatgtgaattttttatcatgttttttaaatttcaaattttaaaaaagttcggatttga

aaaatggtcaaatttgaaaatatccaaatttttaaaaactgaaaaaaaagatataggaat

atagaaagtgaaaaggaaaaagaaagagaaaaacagtaacacaaaccgggaaaccaaaaa

ccagtagcgaaaaccagaaaccgggaaaaccacggtgagccgaactgaaatgttctagaa

ccttctatatatccttgtgcttggtttgtgcaaccatcgaacatgcattggcagcagctg

gcccattcagtctgttcttatctaagaagtctaaaatctaacaagcaggagagctcatga

cgttagcagcagggccgttcgatctgcctctcatcagcatcacgctacgcgtttctttcg

acacgaaccgaaaaggtcagcccggccggccaactcaatgtcccggccggccgccgccga

cgtcaactgttaactagggagctgcgtcaacggaggtaactgtcacatttgcggctcatc

taccgccgcctaccggtactctcgttgctgtccatacgtcgagacgtcgggtgtctcaaa

atggatacgtgactcgtcccgagggatgcccgcgccgccggcgtcgtctccggcgccggc

gcgtccgcgcatgcattttctcctcctctacggcccgcacggcgcacgctccctcacgtc

cattcttcccgatcccgacgcggaaatggtaatctaatccccggcggcgcgcgcgcggct

cgctagtcttccaccactctcggcgcacgctccatgcgatcaatcaatctcccgccgctg

actcgccggctctcccacgctcctacacgttcgacagtcgacacagtgccgactaaaaat

cagttgcgtaacgcacgcggtgagctgaggagaagacggggttaagctggaccttgttgc

caaatactactcccttcgtcgaagagacgccgtgtctacagtacacgcgctccggaacgc

tccaaatggaaggccgccgccgtgtctgctccggccaccagctagctttaccttagctgc

agccatcatctacccgtctcccccaagtcaagctgtagcacacacacatcagtcggtttc

tgcacagcatcgttcccctttcacgcaaaccgcagaacgctagcttccaactcgacgcca

catttgtccacacgtccacatccttaacgccatggatcgctgtcaagaatcgttccatgg

ctaccgatcatgcatcggcgcggccgcgttagtttgactgcctctatgatttcggtactt

aactaaagtacgtacgcgcacctctctgcattttcctagcctccgtacgtacattgcctg

tgatcatcggcaactaacgaactcgatcgtcacggcctccgcaccacgataccacgacgt

cagaacatcgaccggcggca

>DG3C00088.1 - Up_Stream_Len 1700

gctaaataaaagatgtctgtatttatctctgatttgaatatgtacaagtttattacccac

tcccaaataaaaatgtttagcataaaaataagtcaaggggcggatacaagattttcataa

aagttatattattttcaatatgaagtgaagtcctagatcaaacattcctagggtagtatt

gggctcatgatgagaattgaagaattattatcacaattttttaccctattctttgactca

aatagtgatctgccactcggacgctaattttgggctctcaaatgtgtctggattttataa

aaaatgcatgtatatattgaactaattttaaaaattatgagataaatcatgcatgtagag

aatatatgcaggtatgtgcgtatcaaattcgaatctaacatttaaatgtatgtaacctac

acataaaaaacaaattatacgatttataatataagcagtgtacgtatactattcacacta

tactaaagttttattttgtctcttttgcgtaggtcacaaacggtcatattttttctggaa

aatttgcacgagtaggtatcaagctgacatgtacgtgcatgaattatttcaattttaaaa

catttaaaattgcatattcgaattttttgaaaacagttgtaccaataacgaattctctac

ctttttttttcttgtcgctcgtcctaataagcttaaaccgaatttgaccaacaggtcaac

tttcctagcatgtgggtctcatgatagtgacatactgctccttccaagaagctcaattct

agggagcttagagctcttagaggattacctagaggagtgttttgtctttgatatgccaat

ggttcctttatttaaatgtaataagaaaataattttaaatacaaaaaagttttaaaaaat

atgccgcgtagacgacttcatacactgtgcacaccaagtgattttttgtgtggcctatgt

gaaagttaaaatttgttgctaaaaagaagggtttgcacgagacatgtttttgtattgtta

attgttatagagagcaccaaaattgacgttttttgttcgcaaaacttcacatacgcacct

aaaatgtggatatatacgcgagacatatgtttttttacaattaaaatgtgttatttggat

gaaaagggagcagatgcacccgggatcaaaagtaagtttccaaacattacctagtcaaca

atacttgtccatgcatacctatctattcaccctgttgcaatgtcttgttcttataaatcc

gaggcatttatcaaaacggttatgtaaatagaacgtgtgatcaacaaaagctggctggtg

atgttttgtacgtcctgttcttggctctccggcgtaaccggcacggcgggcaggacgact

acagcctcccctcaagcatctctcaagtgattaagcctcggcgactgaccgacgacttca

ggtttggcagatgtaaccatctagctagctactccaccacacttataaattctggactcc

acagatcaggtttgtgatagtcaccggtcagtcgttgcatggcctgactagctcgtggaa

agaatcacggagagctctatgtatcgctctccaggcccttataattagtgagtgtttcgt

tgcaccggagaaaacatgtactcgctcttggtacagcaacggaactgaaacccggccgga

ctttcctagccaccaccacc

>DG5C01302.1 + Up_Stream_Len 1700

gctacattcaaacaaaagggagtaaatttgccaagatggaatactgtgatgagtgaggcc

acagccagaagtattgattgcagaaatgagcaagtatccttgcgcaaggttccacgtaga

gacacaagccttgaataatacacctcgatgcgacgggcataacaaggcaaacctttgttt

cagcacagtgcgatgaggacatccctacaccatgtcttccatataacattatggttactt

ggcttcacacagcacatcatttccaacttctctgcctgcagattctgtggtgcagttttg

cacgctatatacatgctatatactggcctttatgttctctccattgtgattgagctcaga

gactctccaagtggtgtttccactaaaactaaacaatctccagcagtgatgtccctgtct

aatacccagctaatacacatctgctccttcaaggtcactagtttccatcatgttcatcta

tcgacttgctgaaacggcgttcgaaggcagtggcagagcttcagcccactagcctgggca

gctgcccgggctatgcgttgtatcacttgttgaataattagcatgtagtgaaagattagt

cacggttttgattggcaaatggccatggtctacctcagtctttctgctgggtgtgatctt

gtcttgtatggtcgggtgtttcggctaactgttctttttatcaatgcacgaaacgcaagc

tttaagtttctcgaaaaaactaaagacatgttgaagatcaagatatctttttttgtaagc

tagagggaagagtagtagcagctgaataggacatcgattgtctgtatgtgaagatattta

acacaccaacttgtcaccggtaggtaggctggccgtgttcgtagaagctaccaaattaga

atcagtcactgcctccctgggtttttgtggttaggaggaaacgaaagccagagcaatacg

ttgaattgaccagctacctagcacaactaagccatcatgtatgtacttgcgagtctaaac

tcgaaggatcatcgcatcttctctggctagtctactctcgcctccgcacgcagtaaccac

ctccactcgtggccccgtggatcgtccctgtcctctccgcggttagtctccaactcaggt

gagccttctcgatgaccaagttccaaatccattttctctgcgcatccgaaaagaaatact

ccatgtgggattcctccatcagccacaacatgaaagatcgtttagaattgttttaaacgg

attacaagatcgaatctaggccattacggacatgtctatgagaattttttcctagtattg

ttgctactttgcttgcgttcaagctaatcttcgctgcaggagctgtatgtatgtatgctc

tgtgaataatatgttgcgaattcttgcagcaccaagaacaacgataaggaattcttcaga

aaaaataaccatccgacacctctgaagaagcggcaatgaaaagaggattgctttgagagg

ttctgcttatttcactctttcagttgtggtacaaagtgtacaagtattttggcaggtgct

tgttactttaaaaaatgcattgatagtttcattaatgcatcacttgattaatcaccttgc

agatggtcatactggacatattgagaaaaaattgcaactgcactaaccaacagtgaggct

ttagcaactctgagaaaata

>DG6C03650.1 - Up_Stream_Len 1700

gcgtcctcgcggacaagccgacaaaaatgcgtcgggccgctgggagcactttttttcctt

gcccggtcaaaatcggaccgattcagacggccggggtccatttgcgtcgggccgttggag

atgccctaagatcatctccaccggagctcctcaaataagcgttgatagaaacgtcggtag

agcgttattaagagcgccaacatatattctcagcttattaggagctctattccacatcgg

cgctctcaatatttgtacatagttctcataatctatgtgccatcggaggcaccagcatcg

ctgctgcagggtctatcaccgccgccggagtggccaacactgcatcgaagcaacatcggg

gcactggtgacactcacgacgttgctacgggtccaacgacatcacctgctcgtggtggcg

gcgctcgtcctcttcgaggtagtcagtgaagcacctcttgccctacccccacgctcctct

cagggtgccagggaggtagggaagactcgacatgatggcattgcgatgacaccaccgctg

ccatccccccatccggtctcacactccgcggcgctgtttcgctgccgccatcaccggcct

cactgctgctaagttgctactgtgagccgcctcatgcgcgtctctctatgctgcctccct

cctatccacggtcgcattcctctcagcaaccatcctgccggctagcctcgtcgcggagtt

tggtctcgaacaatgcgaccaacgccctttgcttgtcggttacaccccgcgactgtctcc

tcgacgtcgaaggcctccaatgccgtgacctcatcatcaagaaaatggccacgcgccatc

tccctaatcctagcccaggttgcctggtaatctgccatttctatgggggacgttgttgtg

ttgttcccttccccggcgacggcaggcctataaatagctgtgccctcggtgggaaccagc

gctccctcgatgggaaccggcacgcccttagtaggagcgctgactattttagtgaacttt

aaattgtgccggtttgatattaaagtcgtcggtgtaagtcaaaatgaaaatcaacgtcta

aataagtctattgaaagggcagggtggatgaaaaatacatcgacatgggacattaaaaaa

aaaatcaatggaaggttaaaaaattttggaaccttttaggaactaaagatgattaagtat

tataattgtataaaaatatcttgtgaggaaattactagtattataccctaggtaaaaaaa

caaatcgacatgcaatatccatgtagtattcacgctatatttgtcatagattttttttac

taggataccagaggaattatttcgaacccaaaatatttttatactagtataatacttgat

catcttttatttctagaagattttgaatttttttcaattttaaaattcctacaaattttg

agagagccaaagtcctcgtctcagagatcctccaatgttcggcttcggcgcttccccacc

cggcagcacgcaaccacgcgcacacatggaggtgggccccacatggggtacttgtcgact

cgagtccaccaggccgcccgttgaccggcagagcctcccgcaaactaaatcccccctctc

caccacctagcgtcggtcgccagtcgccactccccgtccccgcctccccatcccgacggc

gcgctccgtccctccaccaa

>DG6C02319.1 - Up_Stream_Len 1700

agaatacttcaaaagtgaactttgatacacatgagaaaacatacgaattatgtacaccga

gacttacacatgaggtgagacccacgttattttttccttcgaaatatctatgaaatatcc

cattccataagaattccatagaaatataagattcatattccattattccaaacaaacatt

tgaagtggagagcgagaagggaagtggggagacccgaggtcccgagacctcaccgaactc

cattcgcggctcactggcagtctggcacggtggcgccgtagccattcgtccggccggcca

cccatcagcagacgggttgactggggcagttgaccggcaaagtccgtggatttccgcgtc

ccccactcgccatcgccgggccctcccgttcgccccaccgggcggggccgggcccccaca

cgcttagaaaacctcccactttcttttccttccctttgacggacgggcgccgggcggctt

cccctcctctctcctccctccggtcctcactactcctctccccttccgcgcctcgggtcc

tcctccgtgcttcccacgcaggcgacggcggggcgagcttcaggtgagcagcccgccgac

gccgcctcctcctctagatttgttcatcccgtagctgctctcgcctttcttcgtgtggtg

ccaataatctatatttgtttatttatatatacggtctttgcgagctctggtttgtccgct

tgttcaggggttcttgtgggttggtagaggtttcgcggagatgaagagtgtgtggattag

gaggaggaagtttttggtgtttagtgttcagtgacgcaatttctggagaggaactccggt

ctccactctccaggccctgttgattggtgggggtttgttgcttgctggaggaggagcagc

agcagcaaggttctgcccagtgcttttttttttgttaaaagaaggccgctccgtaggtgt

gaaatccgggagaaagatagtatgctatggacggtgcggtgccattatttcgagagatta

tttctccccttcttagttagatcttcctctttctggaaagtagggccccttttggccgtt

cgagccgttcggctctggcatgagaaatcgcgatgttaaacagtatgaaagccgtttggt

tatcccctggaaataactgatgcctcgattgatggtccaggagccatcgtactgttattc

ttaggaacgatgcaaactgcacttattctctgatgctaaccttagcagaatataggcatc

gtttgtcatttcctatgtctatagtggggatcagttctcctccactgatggtgtccgcgt

ggtattgataccccttctgctttccggtgttgcaatcaagtcaaaatttccatttcagaa

tacggttatcactaagggtgggaaaaaactgaaaccgtgggaaatcgtggaaaccggcta

gtgagagctagttcattttccatcacaaattaaattctcactcccagttgaactagccta

aaaccggttaaaccatgacaccgtgttttaccaccctttgttatcaccaactagcctaaa

accggttaaaccgtgacaccgtatttaatttgactatgttacatgttttttttaacggat

gtttacatgtaggtgaagcttgaacttacaagaggtgttgctcacgaaggacaggctata

ttaaatcatataagtgctct

>DG6C01662.1 - Up_Stream_Len 1700

aggcgggcatctgactctgtgttcaaatttttggcctttttgcagcagtggtatccgctc

tgtaggcagcgggatagggaacgactggacagcatgctggaggatcttctattggcggca

cgccaattatctacgcagtccagccgttgagttgctctacacgtcacgtgggagctgtat

ctttcttctttatcttgttgggcatgcttgtgctgtggccccagcaaacctttttcgctt

ttgctatgttgggtacttgaactctgtatggttgtggttgctttatttataaagcggggc

gaaagcctgtttcgtcgacaacctcatatagcagtcagcacaaaggggcaaatctatact

aattttccaggaacaaggctacacgactccggtgcgtgcacctccgttatctcacgttcc

gttccaacaaccattctattccattgacctcccagaagaaacagtgtactgaacaagtca

gatcataccccaaaccggatatacagattatttttctgtagtagtacactgttcctagaa

aacctgcctagacccgtgtgaatcgtaacactacctacccctcgcgttttttctctcgtg

acgttttcgttgcatatcgggaatacccgggatatcgtggctgccagggagcggaatctt

tggtgtttgttaggatttgactctccgagttagccagttttttatttgctcacgatggca

accagactgtcagatgtaaaccctgagtgaaacacgtgcattcacccaaaacactagaag

taatactctgaattacaccataaaacctgtctaccgaacgagcccttagcgatctttgct

ctacgacggtagatgacaagccgctaaaaaagaactgtaaaaagaagacggtagtagata

acaagcagtcaaaaataaactgtaaaaagaagagcagcccctagcaaacctcagcagcca

gcacaaagggacaaatctatactgattttccaggaactatcaggttacacgactccggtg

cgtgcacctccgttatctcacgctccgttccaacaaccattccattccattgacctccat

aaaaaacagtgcactaacaagtcagatcataccccaaaccggatatacagattatttttc

tgcagtagtacactgtttctcagcttggatcctctgtggaccgaccgacccagcggacgc

cgccatttcgttttgaacgattccgttcctccttcgcgtgcgccaccacgccaccaccac

cacgtcggaagattacgcgtcccaccgtcaccgccaccaccaccattcacaccgcttctc

cctccctctcgccatcgcctcctcccccctcttctccccttcgtcaccgccgatagctac

tcccaagcaatacttttcttcggatcccccgagattaggcacgttctcgcggcagcgggg

cgatttggtcgcccgtttgttccttgattctttcctggcggctgttccgctggttttcct

ctctttctcactccccgcgctcaatccgctccgcgtttcttgccccagtcagcccgcgtt

ctcgtctccagtcgcccgagcgcgcagcaaagatcttggcgccagttcttggaagaggag

gaacatcgccaccgcgccagcccggcgcttcttcggagtcgcggccgtcctgtttcccca

cgaagccgccgctttcgggg

>DG7C03652.1 + Up_Stream_Len 1700

agcctatatgtttcctaacacaatgtgtgctggaaagaataggtaactgaaaaaactggc

cctgcaggtccattaacataggaaaattttggtccagctcagttaacaataatgttttga

aaaatggctaacgaaacaatgttgattgttcatgcggcaagctcgtaaggaagaactagc

aacacagggatctcagagtaaatagtctatatattagaggtccttttgtactaaatagaa

aaaaatactgaagggcttctttgcaatgtaaacaactagctgcacagatcttgaacataa

aatggacgcccaggataagcccgggacgcccacatatatgtagagtgggattttcgaaaa

tattatgcattgcaagttggcaacaagcaaagcgacgcgagcaggtctaacagcaacttt

ctatatgtgaaacctccctttcacagcaagttgaaacatattgtctgttggatgttcaca

ctagatgacatggagcggtactagcggcagaacttctctaacctttggacctttagggtg

tgttcggttctggaactaggggggacggaatgagatggttccatttctagggaatgaaat

ggttccgaccccgtgttcggttcggaaaagaacgaggaacggaatggttccatccctgtg

ttcggttttggaatgggatgagaatggaatggaatggaatgagaatggaattattctaaa

ctcaattaattattcttaatcttaattaattattgctaaacttgattaattattcttaat

cttgattaattatttttaatcttaattaattattgctaaacatgattaattatagcctaa

tcttgattaattattgctaaacttggttaattatagctaaacttgattagcctaatctcc

attccacctcgtccggccgatttgggcggacgtgtgcatttcgcatctgggaggaatatt

ccatttcgaggaactagttggttccgttccttctctccaaccgaacacaggaacgggtcc

taggaacggaaccgacctgttccgttcctcctaattccggaaccgaacacaccagattat

aaggtcatgagacaagctcaatacctaatagtactattaatactaacattctctcagaaa

aaattagcagtagtagtatttaggtgtttaacccattgaactgcaaaatgggtaatctgt

atttcttaattcatgcatcacgtagtttggagtccaagctccgccactaaggggtgcctt

tgtatgtgacaagtggaagtaaaaagattcggttagttggaaggaaaatatgaatgcatt

cagacgtaaaatttgaccggaagtaaaaaagaagaatgcattccaaactcgtgcttgatt

ggcaacacgcctggttgattattcaacacggaggctgccttcctccacctttcctctcaa

ttccttcgtagcggccaatcattgtactaaattcaccaggggaagcacaaatttctggga

agcaaatcaagccgcctttccttccattttgccacccctgcagtcccatcgtgttgttgc

gagctcatctttgcactggtccggcaaactgccgtgagctacagcgcacctttgctatct

tgtttcgttgagacgcagttgttcctccacaaaactgcccaagcagatccgcagtcatcc

gtctacaaaagagtctaatc
