# Supplementary material for: The whole-genome and expression profile analysis of WRKY and RGAs in Dactylis glomerata showed that DG6C02319.1 and DgWRKYs may cooperate in the immunity against rust
Source: PeerJ. 2021 Aug 19;9:e11919. doi: 10.7717/peerj.11919 (PMC8380429; doi:10.7717/peerj.11919)
Supplement: Supplemental Information 5 [file peerj-09-11919-s005.docx]

**Table S5:** The conserved domain and location of all DgWRKY sequences.

| seq id | alignment  start | alignment  end | envelope  start | envelope  end | hmm  acc | hmm  name | hmm  start | Hmm  end | Hmm  length |
| --- | --- | --- | --- | --- | --- | --- | --- | --- | --- |
| >DgWRKY83.1 | 104 | 160 | 104 | 161 | PF03106.15 | WRKY | 1 | 58 | 59 |
| >DgWRKY28.1 | 118 | 175 | 118 | 176 | PF03106.15 | WRKY | 1 | 58 | 59 |
| >DgWRKY27.1 | 167 | 225 | 167 | 225 | PF03106.15 | WRKY | 1 | 59 | 59 |
| >DgWRKY26.1 | 167 | 225 | 167 | 225 | PF03106.15 | WRKY | 1 | 59 | 59 |
| >DgWRKY48.1 | 130 | 187 | 130 | 187 | PF03106.15 | WRKY | 1 | 59 | 59 |
| >DgWRKY81.1 | 202 | 261 | 202 | 262 | PF03106.15 | WRKY | 1 | 58 | 59 |
| >DgWRKY85.1 | 198 | 253 | 197 | 253 | PF03106.15 | WRKY | 2 | 59 | 59 |
| >DgWRKY85.1 | 366 | 422 | 365 | 422 | PF03106.15 | WRKY | 2 | 59 | 59 |
| >DgWRKY45.1 | 163 | 220 | 162 | 220 | PF03106.15 | WRKY | 2 | 59 | 59 |
| >DgWRKY50.1 | 152 | 211 | 152 | 212 | PF03106.15 | WRKY | 1 | 58 | 59 |
| >DgWRKY10.1 | 321 | 377 | 321 | 377 | PF03106.15 | WRKY | 1 | 59 | 59 |
| >DgWRKY10.1 | 551 | 608 | 551 | 608 | PF03106.15 | WRKY | 1 | 59 | 59 |
| >DgWRKY32.1 | 296 | 353 | 295 | 353 | PF03106.15 | WRKY | 2 | 59 | 59 |
| >DgWRKY32.1 | 247 | 292 | 247 | 292 | PF10533.9 | Plant_zn_clust | 1 | 49 | 49 |
| >DgWRKY82.1 | 246 | 306 | 246 | 306 | PF03106.15 | WRKY | 1 | 59 | 59 |
| >DgWRKY7.1 | 237 | 294 | 236 | 294 | PF03106.15 | WRKY | 2 | 59 | 59 |
| >DgWRKY89.1 | 160 | 217 | 159 | 218 | PF03106.15 | WRKY | 2 | 58 | 59 |
| >DgWRKY44.1 | 110 | 166 | 110 | 167 | PF03106.15 | WRKY | 1 | 58 | 59 |
| >DgWRKY58.1 | 99 | 156 | 98 | 157 | PF03106.15 | WRKY | 2 | 58 | 59 |
| >DgWRKY62.1 | 63 | 123 | 62 | 123 | PF03106.15 | WRKY | 2 | 59 | 59 |
| >DgWRKY60.1 | 127 | 189 | 126 | 189 | PF03106.15 | WRKY | 2 | 59 | 59 |
| >DgWRKY57.1 | 222 | 277 | 222 | 278 | PF03106.15 | WRKY | 1 | 58 | 59 |
| >DgWRKY57.1 | 392 | 449 | 392 | 449 | PF03106.15 | WRKY | 1 | 59 | 59 |
| >DgWRKY61.1 | 115 | 177 | 114 | 178 | PF03106.15 | WRKY | 2 | 58 | 59 |
| >DgWRKY59.1 | 99 | 156 | 98 | 157 | PF03106.15 | WRKY | 2 | 58 | 59 |
| >DgWRKY63.1 | 56 | 115 | 55 | 116 | PF03106.15 | WRKY | 2 | 58 | 59 |
| >DgWRKY46.1 | 103 | 163 | 102 | 164 | PF03106.15 | WRKY | 2 | 58 | 59 |
| >DgWRKY86.1 | 233 | 289 | 232 | 290 | PF03106.15 | WRKY | 2 | 58 | 59 |
| >DgWRKY86.1 | 196 | 229 | 188 | 229 | PF10533.9 | Plant_zn_clust | 16 | 49 | 49 |
| >DgWRKY84.1 | 190 | 431 | 188 | 439 | PF00931.22 | NB-ARC | 4 | 252 | 252 |
| >DgWRKY84.1 | 1017 | 1079 | 1016 | 1079 | PF03106.15 | WRKY | 2 | 59 | 59 |
| >DgWRKY84.1 | 16 | 99 | 12 | 138 | PF18052.1 | Rx_N | 5 | 85 | 93 |
| >DgWRKY14.1 | 156 | 212 | 155 | 213 | PF03106.15 | WRKY | 2 | 58 | 59 |
| >DgWRKY64.1 | 147 | 202 | 146 | 203 | PF03106.15 | WRKY | 2 | 58 | 59 |
| >DgWRKY47.1 | 258 | 315 | 257 | 315 | PF03106.15 | WRKY | 2 | 59 | 59 |
| >DgWRKY66.1 | 10 | 67 | 10 | 67 | PF03106.15 | WRKY | 1 | 59 | 59 |
| >DgWRKY72.1 | 147 | 203 | 147 | 204 | PF03106.15 | WRKY | 1 | 58 | 59 |
| >DgWRKY73.1 | 136 | 193 | 136 | 193 | PF03106.15 | WRKY | 1 | 59 | 59 |
| >DgWRKY34.1 | 41 | 101 | 41 | 102 | PF03106.15 | WRKY | 1 | 58 | 59 |
| >DgWRKY37.1 | 322 | 379 | 321 | 379 | PF03106.15 | WRKY | 2 | 59 | 59 |
| >DgWRKY37.1 | 274 | 318 | 274 | 318 | PF10533.9 | Plant_zn_clust | 1 | 49 | 49 |
| >DgWRKY36.1 | 141 | 197 | 141 | 198 | PF03106.15 | WRKY | 1 | 58 | 59 |
| >DgWRKY35.1 | 178 | 234 | 178 | 234 | PF03106.15 | WRKY | 1 | 59 | 59 |
| >DgWRKY35.1 | 312 | 369 | 312 | 369 | PF03106.15 | WRKY | 1 | 59 | 59 |
| >DgWRKY17.1 | 114 | 172 | 113 | 173 | PF03106.15 | WRKY | 2 | 58 | 59 |
| >DgWRKY29.1 | 286 | 342 | 286 | 342 | PF03106.15 | WRKY | 1 | 59 | 59 |
| >DgWRKY29.1 | 502 | 559 | 502 | 559 | PF03106.15 | WRKY | 1 | 59 | 59 |
| >DgWRKY79.1 | 187 | 244 | 186 | 244 | PF03106.15 | WRKY | 2 | 59 | 59 |
| >DgWRKY53.1 | 149 | 206 | 149 | 206 | PF03106.15 | WRKY | 1 | 59 | 59 |
| >DgWRKY52.1 | 147 | 204 | 147 | 204 | PF03106.15 | WRKY | 1 | 59 | 59 |
| >DgWRKY31.1 | 239 | 297 | 239 | 297 | PF03106.15 | WRKY | 1 | 59 | 59 |
| >DgWRKY6.1 | 195 | 425 | 178 | 427 | PF00931.22 | NB-ARC | 18 | 250 | 252 |
| >DgWRKY6.1 | 12 | 98 | 12 | 133 | PF18052.1 | Rx_N | 1 | 86 | 93 |
| >DgWRKY6.1 | 892 | 950 | 892 | 952 | PF03106.15 | WRKY | 1 | 57 | 59 |
| >DgWRKY8.1 | 155 | 211 | 155 | 212 | PF03106.15 | WRKY | 1 | 58 | 59 |
| >DgWRKY39.1 | 270 | 326 | 269 | 327 | PF03106.15 | WRKY | 2 | 58 | 59 |
| >DgWRKY39.1 | 220 | 266 | 220 | 266 | PF10533.9 | Plant_zn_clust | 1 | 49 | 49 |
| >DgWRKY56.1 | 78 | 134 | 77 | 135 | PF03106.15 | WRKY | 2 | 58 | 59 |
| >DgWRKY55.1 | 155 | 193 | 149 | 193 | PF03106.15 | WRKY | 20 | 59 | 59 |
| >DgWRKY54.1 | 81 | 138 | 80 | 138 | PF03106.15 | WRKY | 2 | 59 | 59 |
| >DgWRKY90.1 | 191 | 248 | 191 | 249 | PF03106.15 | WRKY | 1 | 58 | 59 |
| >DgWRKY78.1 | 139 | 199 | 139 | 200 | PF03106.15 | WRKY | 1 | 58 | 59 |
| >DgWRKY77.1 | 107 | 166 | 107 | 167 | PF03106.15 | WRKY | 1 | 58 | 59 |
| >DgWRKY41.1 | 173 | 230 | 172 | 230 | PF03106.15 | WRKY | 2 | 59 | 59 |
| >DgWRKY12.1 | 309 | 367 | 309 | 367 | PF03106.15 | WRKY | 1 | 59 | 59 |
| >DgWRKY49.1 | 203 | 259 | 203 | 260 | PF03106.15 | WRKY | 1 | 58 | 59 |
| >DgWRKY25.1 | 125 | 184 | 125 | 185 | PF03106.15 | WRKY | 1 | 58 | 59 |
| >DgWRKY24.1 | 126 | 185 | 126 | 186 | PF03106.15 | WRKY | 1 | 58 | 59 |
| >DgWRKY23.1 | 107 | 166 | 107 | 167 | PF03106.15 | WRKY | 1 | 58 | 59 |
| >DgWRKY21.1 | 129 | 193 | 128 | 193 | PF03106.15 | WRKY | 2 | 59 | 59 |
| >DgWRKY20.1 | 134 | 198 | 133 | 198 | PF03106.15 | WRKY | 2 | 59 | 59 |
| >DgWRKY74.1 | 163 | 220 | 162 | 220 | PF03106.15 | WRKY | 2 | 59 | 59 |
| >DgWRKY65.1 | 133 | 189 | 133 | 190 | PF03106.15 | WRKY | 1 | 58 | 59 |
| >DgWRKY0-1.1 | 237 | 294 | 236 | 294 | PF03106.15 | WRKY | 2 | 59 | 59 |
| >DgWRKY9.1 | 176 | 232 | 176 | 232 | PF03106.15 | WRKY | 1 | 59 | 59 |
| >DgWRKY9.1 | 341 | 398 | 341 | 398 | PF03106.15 | WRKY | 1 | 59 | 59 |
| >DgWRKY18.1 | 33 | 94 | 33 | 94 | PF03106.15 | WRKY | 1 | 59 | 59 |
| >DgWRKY33.1 | 231 | 287 | 231 | 287 | PF03106.15 | WRKY | 1 | 59 | 59 |
| >DgWRKY33.1 | 391 | 448 | 391 | 448 | PF03106.15 | WRKY | 1 | 59 | 59 |
| >DgWRKY0-2.1 | 119 | 179 | 119 | 179 | PF03106.15 | WRKY | 1 | 59 | 59 |
| >DgWRKY0-3.1 | 119 | 179 | 119 | 179 | PF03106.15 | WRKY | 1 | 59 | 59 |
| >DgWRKY16.1 | 207 | 263 | 207 | 263 | PF03106.15 | WRKY | 1 | 59 | 59 |
| >DgWRKY16.1 | 368 | 425 | 368 | 425 | PF03106.15 | WRKY | 1 | 59 | 59 |
| >DgWRKY43.1 | 252 | 310 | 252 | 310 | PF03106.15 | WRKY | 1 | 59 | 59 |
| >DgWRKY40.1 | 333 | 389 | 332 | 390 | PF03106.15 | WRKY | 2 | 58 | 59 |
| >DgWRKY40.1 | 291 | 329 | 284 | 329 | PF10533.9 | Plant_zn_clust | 9 | 49 | 49 |
| >DgWRKY38.1 | 3 | 58 | 3 | 59 | PF03106.15 | WRKY | 1 | 58 | 59 |
| >DgWRKY68.1 | 89 | 148 | 89 | 149 | PF03106.15 | WRKY | 1 | 58 | 59 |
| >DgWRKY67.1 | 305 | 362 | 304 | 362 | PF03106.15 | WRKY | 2 | 59 | 59 |
| >DgWRKY2.1 | 173 | 227 | 173 | 228 | PF03106.15 | WRKY | 1 | 58 | 59 |
| >DgWRKY2.1 | 338 | 394 | 338 | 395 | PF03106.15 | WRKY | 1 | 58 | 59 |
| >DgWRKY3.1 | 229 | 285 | 229 | 285 | PF03106.15 | WRKY | 1 | 59 | 59 |
| >DgWRKY3.1 | 402 | 459 | 402 | 459 | PF03106.15 | WRKY | 1 | 59 | 59 |
| >DgWRKY87.1 | 199 | 247 | 192 | 247 | PF03106.15 | WRKY | 18 | 59 | 59 |
| >DgWRKY88.1 | 96 | 160 | 95 | 160 | PF03106.15 | WRKY | 2 | 59 | 59 |
| >DgWRKY1.1 | 122 | 182 | 122 | 182 | PF03106.15 | WRKY | 1 | 59 | 59 |
| >DgWRKY5.1 | 245 | 301 | 244 | 302 | PF03106.15 | WRKY | 2 | 58 | 59 |
| >DgWRKY5.1 | 196 | 240 | 196 | 241 | PF10533.9 | Plant_zn_clust | 1 | 48 | 49 |
| >DgWRKY4.1 | 124 | 180 | 124 | 181 | PF03106.15 | WRKY | 1 | 58 | 59 |
| >DgWRKY11.1 | 262 | 318 | 261 | 319 | PF03106.15 | WRKY | 2 | 58 | 59 |
| >DgWRKY11.1 | 216 | 258 | 216 | 258 | PF10533.9 | Plant_zn_clust | 1 | 49 | 49 |
| >DgWRKY51.1 | 181 | 238 | 181 | 238 | PF03106.15 | WRKY | 1 | 59 | 59 |
| >DgWRKY13.1 | 190 | 247 | 190 | 248 | PF03106.15 | WRKY | 1 | 58 | 59 |
| >DgWRKY69.1 | 186 | 243 | 186 | 243 | PF03106.15 | WRKY | 1 | 59 | 59 |
| >DgWRKY70.1 | 155 | 212 | 155 | 213 | PF03106.15 | WRKY | 1 | 58 | 59 |
| >DgWRKY71.1 | 145 | 202 | 145 | 203 | PF03106.15 | WRKY | 1 | 58 | 59 |
| >DgWRKY22.1 | 375 | 430 | 374 | 430 | PF03106.15 | WRKY | 2 | 59 | 59 |
| >DgWRKY22.1 | 532 | 584 | 530 | 585 | PF03106.15 | WRKY | 3 | 58 | 59 |
| >DgWRKY30.1 | 272 | 328 | 272 | 328 | PF03106.15 | WRKY | 1 | 59 | 59 |
| >DgWRKY30.1 | 485 | 542 | 485 | 542 | PF03106.15 | WRKY | 1 | 59 | 59 |
| >DgWRKY80.1 | 195 | 252 | 195 | 252 | PF03106.15 | WRKY | 1 | 59 | 59 |
| >DgWRKY80.1 | 352 | 409 | 352 | 409 | PF03106.15 | WRKY | 1 | 59 | 59 |
| >DgWRKY15.1 | 98 | 155 | 98 | 155 | PF03106.15 | WRKY | 1 | 59 | 59 |
| >DgWRKY19.1 | 153 | 209 | 153 | 210 | PF03106.15 | WRKY | 1 | 58 | 59 |
| >DgWRKY76.1 | 138 | 198 | 138 | 199 | PF03106.15 | WRKY | 1 | 58 | 59 |
| >DgWRKY75.1 | 111 | 170 | 111 | 171 | PF03106.15 | WRKY | 1 | 58 | 59 |
| >DgWRKY42.1 | 182 | 238 | 182 | 239 | PF03106.15 | WRKY | 1 | 58 | 59 |
