# Supplementary material for: The whole-genome and expression profile analysis of WRKY and RGAs in Dactylis glomerata showed that DG6C02319.1 and DgWRKYs may cooperate in the immunity against rust
Source: PeerJ. 2021 Aug 19;9:e11919. doi: 10.7717/peerj.11919 (PMC8380429; doi:10.7717/peerj.11919)
Supplement: Supplemental Information 6 [file peerj-09-11919-s006.docx]

**Table S6:** The chromosome locations, WRKY domains, zinc finger motifs and gene lengths of all DgWRKY sequences.

| Gene name | Group | Chromosome location | Gene length | WRKY domain | Zinc finger |
| --- | --- | --- | --- | --- | --- |
| DgWRKY10 | Ⅰ | Chr1: 226040935-226044683 | 2723 | WRKYGQK×2 | CX4–5CX22–23HX1H |
| DgWRKY29 | Ⅰ | Chr3: 185829700-185832898 | 2233 | WRKYGQK×2 | CX4–5CX22–23HX1H |
| DgWRKY30 | Ⅰ | Chr3: 218469765-218472233 | 2001 | WRKYGKR, WRKYGQK | CX4–5CX22–23HX1H |
| DgWRKY3 | Ⅰ | Chr1: 66712644-66720298 | 2345 | WRKYGQK×2 | CX4–5CX22–23HX1H |
| DgWRKY22 | Ⅰ | Chr3: 127396922-127402222 | 1767 | WRKYGQK×2 | CX4–5CX22–23HX1H |
| DgWRKY57 | Ⅰ | Chr5: 186357065-186359444 | 1975 | WRKYGQK×2 | CX4–5CX22–23HX1H |
| DgWRKY85 | Ⅰ | Chr7: 91502087-91510685 | 2024 | WRKYGQK×2 | CX4–5CX22–23HX1H |
| DgWRKY33 | Ⅰ | Chr4: 63788586-63793880 | 2310 | WRKYGQK×2 | CX4–5CX22–23HX1H |
| DgWRKY9 | Ⅰ | Chr1: 210635614-210637708 | 1512 | WRKYGQK×2 | CX4–5CX22–23HX1H |
| DgWRKY16 | Ⅰ | Chr2: 158545275-158553015 | 1461 | WRKYGQK×2 | CX4–5CX22–23HX1H |
| DgWRKY80 | Ⅰ | Chr6: 165446598-165448515 | 1739 | WRKYGQK×2 | CX4–5CX22–23HX1H |
| DgWRKY2 | Ⅰ | Chr1: 61630219-61633680 | 1794 | WRKYGQK×2 | CX4–5CX22–23HX1H |
| DgWRKY35 | Ⅰ | Chr4: 170470500-170473746 | 1239 | WRKYGQK×2 | CX4–5CX22–23HX1H |
| DgWRKY45 | Ⅱa | Chr5: 68632140-68635138 | 2470 | WRKYGQK | CX4–5CX22–23HX1H |
| DgWRKY31 | Ⅱa | Chr3: 236136691-236142957 | 1785 | WRKYGQK | CX4–5CX22–23HX1H |
| DgWRKY12 | Ⅱa | Chr2: 23345546-23347724 | 1662 | WRKYGQK | CX4–5CX22–23HX1H |
| DgWRKY67 | Ⅱa | Chr6: 11037641-11041172 | 1662 | WRKYGQK | CX4–5CX22–23HX1H |
| DgWRKY47 | Ⅱa | Chr5: 82570608-82575411 | 1725 | WRKYGQK | CX4–5CX22–23HX1H |
| DgWRKY43 | Ⅱa | Chr5: 38711495-38713437 | 1503 | WRKYGQK | CX4–5CX22–23HX1H |
| DgWRKY13 | Ⅱa | Chr2: 81553504-81554985 | 1482 | WRKYGQK | CX4–5CX22–23HX1H |
| DgWRKY90 | Ⅱa | Chr7: 201954611-201956004 | 1282 | WRKYGQK | CX4–5CX22–23HX1H |
| DgWRKY27 | Ⅱa | Chr3: 167574487-167575689 | 966 | WRKYGQK | CX4–5CX22–23HX1H |
| DgWRKY26 | Ⅱa | Chr3: 167483992-167485194 | 966 | WRKYGQK | CX4–5CX22–23HX1H |
| DgWRKY28 | Ⅱa | Chr3: 167584252-167585898 | 819 | WRKYGQK | CX4–5CX22–23HX1H |
| DgWRKY51 | Ⅱb | Chr5: 156459672-156461776 | 1140 | WRKYGQK | CX4–5CX22–23HX1H |
| DgWRKY49 | Ⅱb | Chr5: 142977297-142979101 | 1041 | WRKYGQK | CX4–5CX22–23HX1H |
| DgWRKY69 | Ⅱb | Chr6: 17414009-17415864 | 1155 | WRKYGQK | CX4–5CX22–23HX1H |
| DgWRKY36 | Ⅱb | Chr4: 170807637-170809463 | 936 | WRKYGQK | CX4–5CX22–23HX1H |
| DgWRKY4 | Ⅱb | Chr1: 155645142-155647665 | 852 | WRKYGQK | CX4–5CX22–23HX1H |
| DgWRKY72 | Ⅱb | Chr6: 23414480-23415483 | 807 | WRKYGKK | CX4–5CX22–23HX1H |
| DgWRKY42 | Ⅱb | Chr5: 38538305-38543621 | 1381 | WRKYGQK | CX4–5CX22–23HX1H |
| DgWRKY48 | Ⅱb | Chr5: 129437241-129438331 | 921 | WRKYGKK | CX4–5CX22–23HX1H |
| DgWRKY53 | Ⅱb | Chr5: 169990647-169993174 | 702 | WRKYGKK | CX4–5CX22–23HX1H |
| DgWRKY8 | Ⅱb | Chr1: 200558822-200563316 | 699 | WRKYGQK | CX4–5CX22–23HX1H |
| DgWRKY52 | Ⅱb | Chr5: 169979286-169980980 | 696 | WRKYGKK | CX4GX23HX1H |
| DgWRKY19 | Ⅱb | Chr3: 24697945-24699947 | 690 | WRKYGQK | CX4–5CX22–23HX1H |
| DgWRKY66 | Ⅱb | Chr6: 9402782-9403528 | 654 | WRKYGQK | CX4–5CX22–23HX1H |
| DgWRKY55 | Ⅱb | Chr5: 181911426-181928792 | 645 | WRKSYYR | CX4–5CX22–23HX1H |
| DgWRKY73 | Ⅱb | Chr6: 26251305-26254666 | 645 | WRKYGQK | CX4–5CX22–23HX1H |
| DgWRKY44 | Ⅱb | Chr5: 42613163-42613798 | 636 | WRKYGKK | CX4–5CX22–23HX1H |
| DgWRKY83 | Ⅱb | Chr6: 186740073-186740888 | 816 | WRKYGKK | CX4–5CX22–23HX1H |
| DgWRKY15 | Ⅱb | Chr2: 149033362-149040937 | 570 | WRKYGQK | CX4–5CX22–23HX1H |
| DgWRKY7 | Ⅱc | Chr1: 191144740-191148331 | 1449 | WRKYGQK | CX4–5CX22–23HX1H |
| DgWRKY0-1 | Ⅱc | Scaffold1464: 16052-19806 | 1398 | WRKYGQK | CX4–5CX22–23HX1H |
| DgWRKY40 | Ⅱc | Chr4: 221921372-221922806 | 1215 | WRKYGQK | CX4–5CX22–23HX1H |
| DgWRKY37 | Ⅱc | Chr4: 175505375-175508485 | 1744 | WRKYGQK | CX4–5CX22–23HX1H |
| DgWRKY89 | Ⅱc | Chr7: 175999445-176001016 | 1479 | WRKYGQK | CX5CX24HX1H |
| DgWRKY41 | Ⅱc | Chr4: 246663868-246666712 | 1244 | WRKYGQK | CX4–5CX22–23HX1H |
| DgWRKY32 | Ⅱc | Chr4: 25650465-25653688 | 2235 | WRKYGQK | CX4–5CX22–23HX1H |
| DgWRKY11 | Ⅱc | Chr1: 231839479-231840916 | 1228 | WRKYGQK | CX4–5CX22–23HX1H |
| DgWRKY79 | Ⅱc | Chr6: 75579286-75580421 | 1020 | WRKYGQK | CX4–5CX22–23HX1H |
| DgWRKY39 | Ⅱc | Chr4: 207087381-207090149 | 1650 | WRKYGQK | CX4–5CX22–23HX1H |
| DgWRKY14 | Ⅱc | Chr2: 112438828-112440020 | 999 | WRKYGQK | CX4–5CX22–23HX1H |
| DgWRKY74 | Ⅱc | Chr6: 37298518-37300076 | 981 | WRKYGQK | CX4–5CX22–23HX1H |
| DgWRKY5 | Ⅱc | Chr1: 161201281-161202643 | 1162 | WRKYGQK | CX4–5CX22–23HX1H |
| DgWRKY54 | Ⅱc | Chr5: 178321332-178323011 | 1450 | WRKYGQK | CX4–5CX22–23HX1H |
| DgWRKY86 | Ⅱc | Chr7: 97991122-97992148 | 1027 | WRKYGQK | CX4–5CX22–23HX1H |
| DgWRKY70 | Ⅱc | Chr6: 17671670-17672712 | 891 | WRKYGQK | CX4–5CX22–23HX1H |
| DgWRKY71 | Ⅱc | Chr6: 17680389-17681470 | 885 | WRKYGQK | CX4–5CX22–23HX1H |
| DgWRKY56 | Ⅱc | Chr5: 182447850-182449116 | 1046 | WRKYGQK | CX4–5CX22–23HX1H |
| DgWRKY65 | Ⅱd | Chr5: 250614852-250617963 | 1535 | WRKYGQK | CX4–5CX22–23HX1H |
| DgWRKY64 | Ⅱd | Chr5: 202271941-202284563 | 969 | WRKYGQK | CX4–5CX22–23HX1H |
| DgWRKY38 | Ⅱd | Chr4: 199597856-199598216 | 285 | WKKYGQK | CX4–5CX22–23HX1H |
| DgWRKY84 | Ⅲ | Chr7: 14601454-14607791 | 5453 | WRKYGQK | CX7CX23HX1C |
| DgWRKY6 | Ⅲ | Chr1: 168091245-168101127 | 3673 | WRKYGQK | CX7CX23HX1Y |
| DgWRKY82 | Ⅲ | Chr6: 175760905-175762990 | 1353 | WRKYGQK | CX7CX23HX1C |
| DgWRKY81 | Ⅲ | Chr6: 167434963-167438253 | 1167 | WRKYGQK | CX7CX23HX1C |
| DgWRKY46 | Ⅲ | Chr5: 79281871-79283204 | 1083 | WRKYGQK | CX7CX25HX1C |
| DgWRKY61 | Ⅲ | Chr5: 187940912-187943624 | 1080 | WKKYGQK | CX7CX27HX1C |
| DgWRKY1 | Ⅲ | Chr1: 20561960-20563900 | 1062 | WRKYGQK | CX7CX23HX1C |
| DgWRKY87 | Ⅲ | Chr7: 151857652-151858765 | 1052 | —— | CX7CX28HX1C |
| DgWRKY50 | Ⅲ | Chr5: 153166616-153168303 | 1523 | WRKYGQK | CX7CX23HX1C |
| DgWRKY24 | Ⅲ | Chr3: 152484638-152486523 | 1432 | WRKYGQK | CX7CX23HX1C |
| DgWRKY20 | Ⅲ | Chr3: 121319007-121320505 | 1014 | WKKYGQK | CX7CX28HX1C |
| DgWRKY21 | Ⅲ | Chr3: 121449133-121450538 | 996 | WKKYGQK | CX7CX28HX1C |
| DgWRKY25 | Ⅲ | Chr3: 152499236-152553902 | 993 | WRKYGQK | CX7CX23HX1C |
| DgWRKY60 | Ⅲ | Chr5: 187818990-187820901 | 948 | WRKYGQK | CX7CX26HX1C |
| DgWRKY0-2 | Ⅲ | Scaffold1608: 1256-2200 | 945 | WRKYGQK | CX7CX23HX1C |
| DgWRKY0-3 | Ⅲ | Scaffold1608: 87533-88477 | 945 | WRKYGQK | CX7CX23HX1C |
| DgWRKY17 | Ⅲ | Chr2: 188974982-188976185 | 936 | WRKYGQK | CX7CX23HX1C |
| DgWRKY23 | Ⅲ | Chr3: 152340230-152341771 | 1124 | WRKYGQK | CX7CX23HX1C |
| DgWRKY75 | Ⅲ | Chr6: 52121991-52122902 | 912 | WRKYGQK | CX7CX23HX1C |
| DgWRKY77 | Ⅲ | Chr6: 52385361-52386263 | 903 | WRKYGQK | CX7CX23HX1C |
| DgWRKY58 | Ⅲ | Chr5: 187668809-187669787 | 900 | WRKYGQK | CX6CX23HX1C |
| DgWRKY78 | Ⅲ | Chr6: 52389647-52392827 | 882 | WRKYGEK | CX7CX24HX1C |
| DgWRKY59 | Ⅲ | Chr5: 187762461-187763452 | 819 | WRKYGQK | CX6CX23HX1C |
| DgWRKY88 | Ⅲ | Chr7: 151871924-151872905 | 807 | WRKYGQK | CX7CX28HX1C |
| DgWRKY62 | Ⅲ | Chr5: 188112758-188116858 | 804 | WRKYGQK | CX7CX24HX1C |
| DgWRKY63 | Ⅲ | Chr5: 188147568-188151366 | 786 | WRKYGQK | CX7CX24HX1C |
| DgWRKY68 | Ⅲ | Chr6: 13294900-13295640 | 741 | WRKYGQK | CX7CX23HX1C |
| DgWRKY76 | Ⅲ | Chr6: 52129101-52131965 | 717 | WRKYGEK | CX7CX24HX1C |
| DgWRKY34 | Ⅲ | Chr4: 143748974-143751943 | 988 | WRKYGEK | CX7CX24HX1C |
| DgWRKY18 | Ⅲ | Chr2: 192543692-192544727 | 672 | WRKYGEK | CX7CX24HX1C |
