# Supplementary figures and images for: The whole-genome and expression profile analysis of WRKY and RGAs in Dactylis glomerata showed that DG6C02319.1 and DgWRKYs may cooperate in the immunity against rust
Source: PeerJ. 2021 Aug 19;9:e11919. doi: 10.7717/peerj.11919 (PMC8380429; doi:10.7717/peerj.11919)

Motif 1

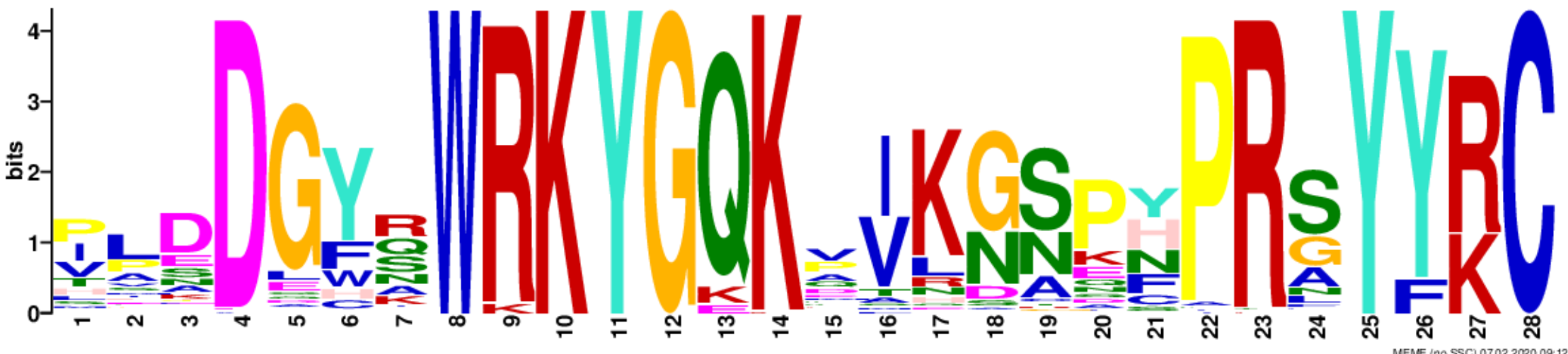

Motif 2

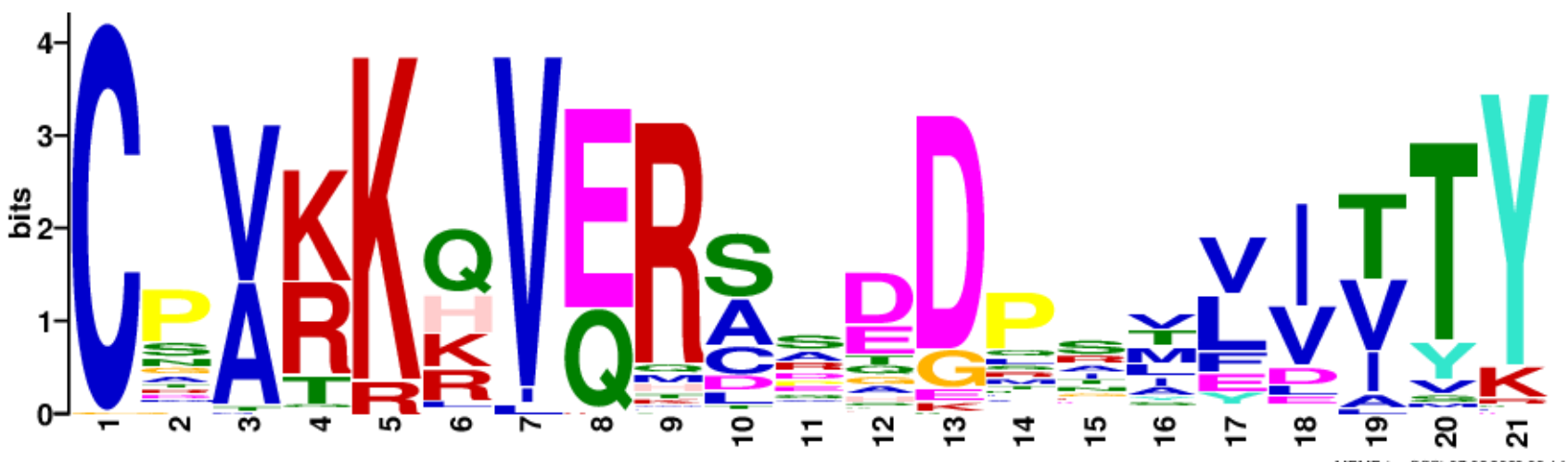

Motif 3

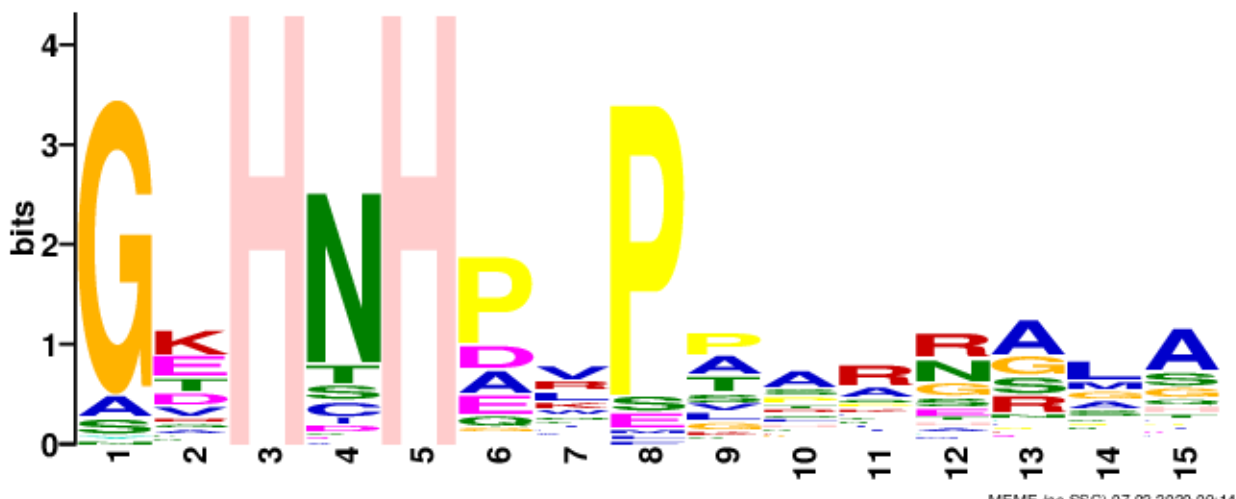

Motif 4

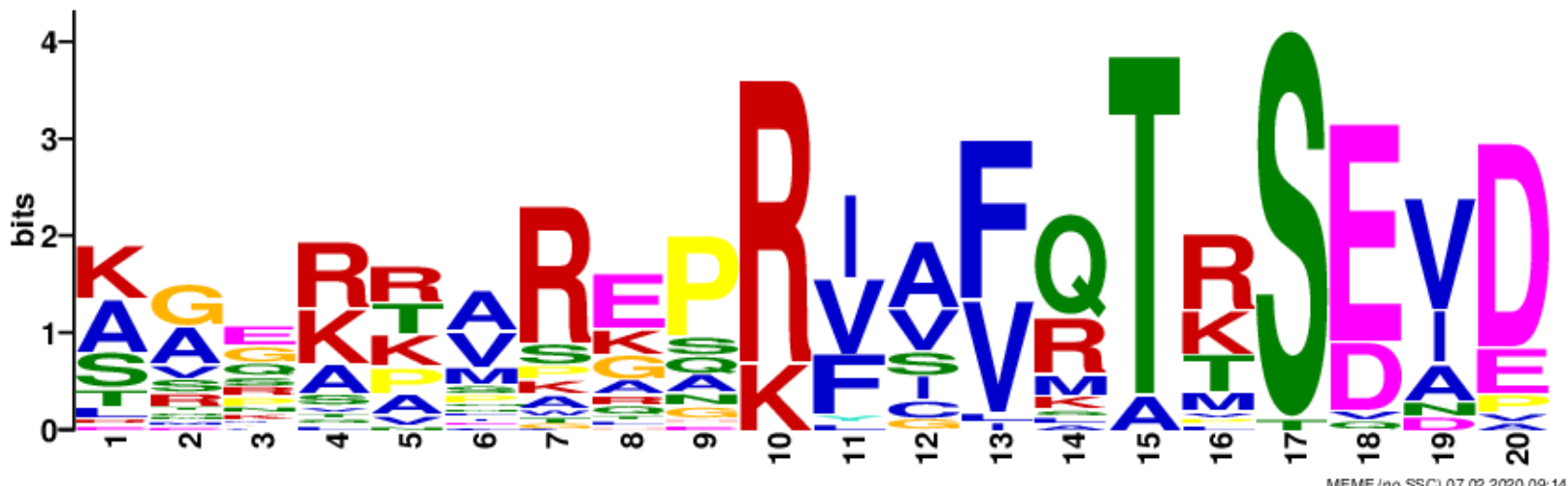

Motif 5

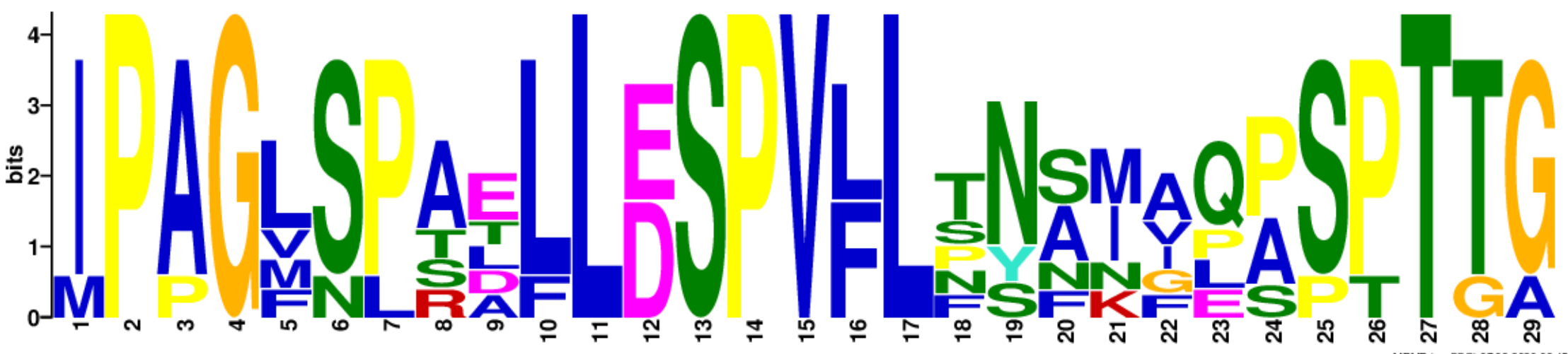

Motif 6

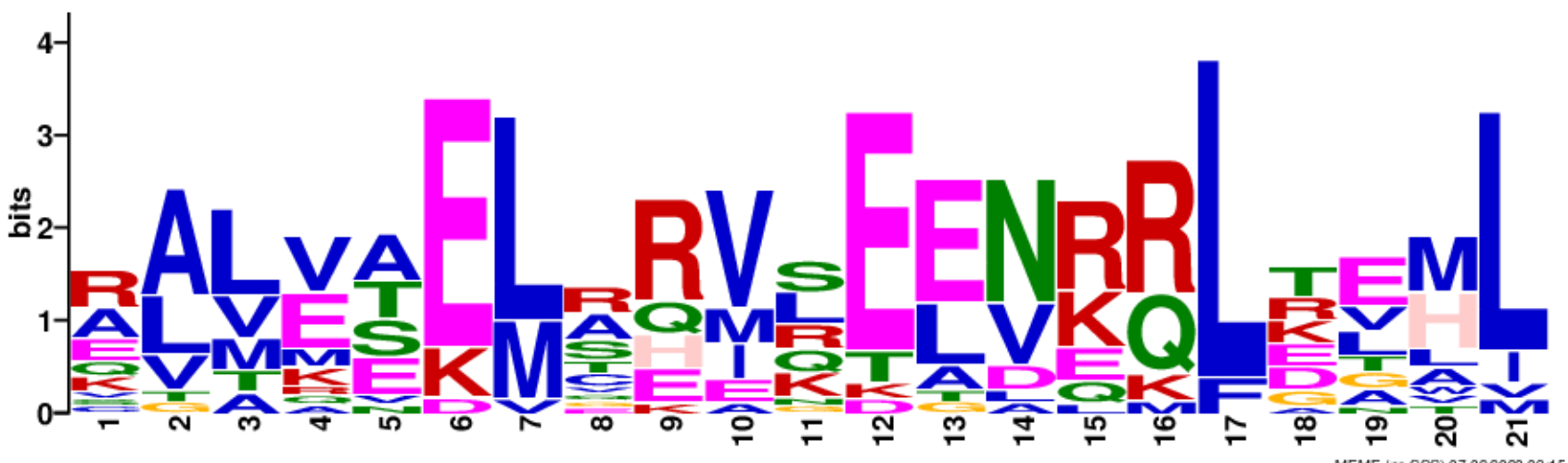

Motif 7

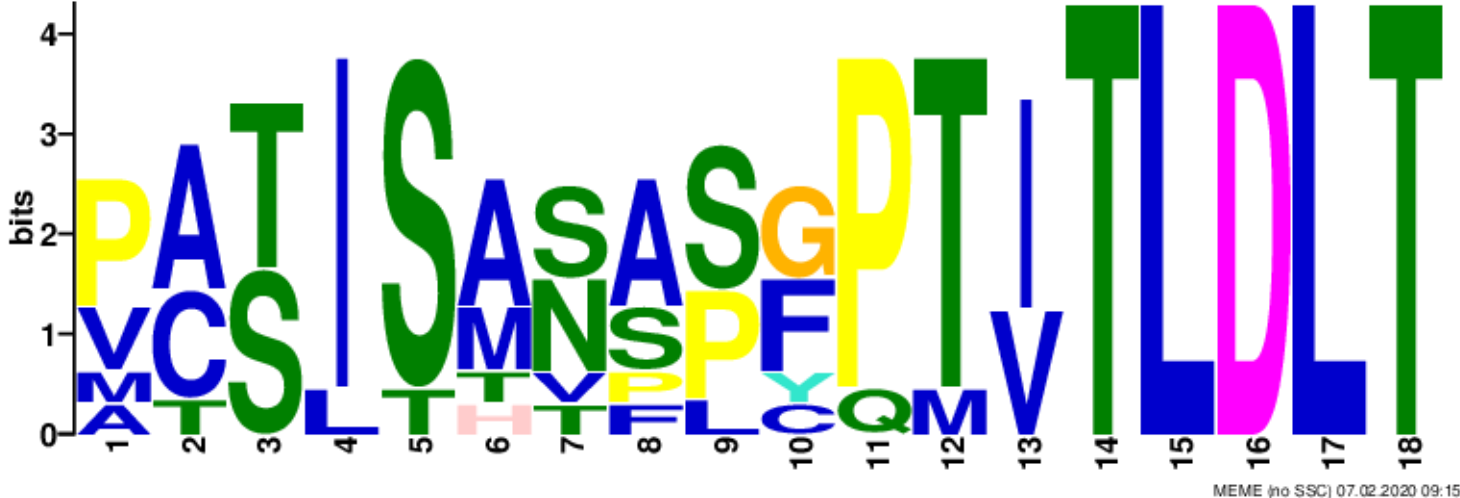

Motif 8

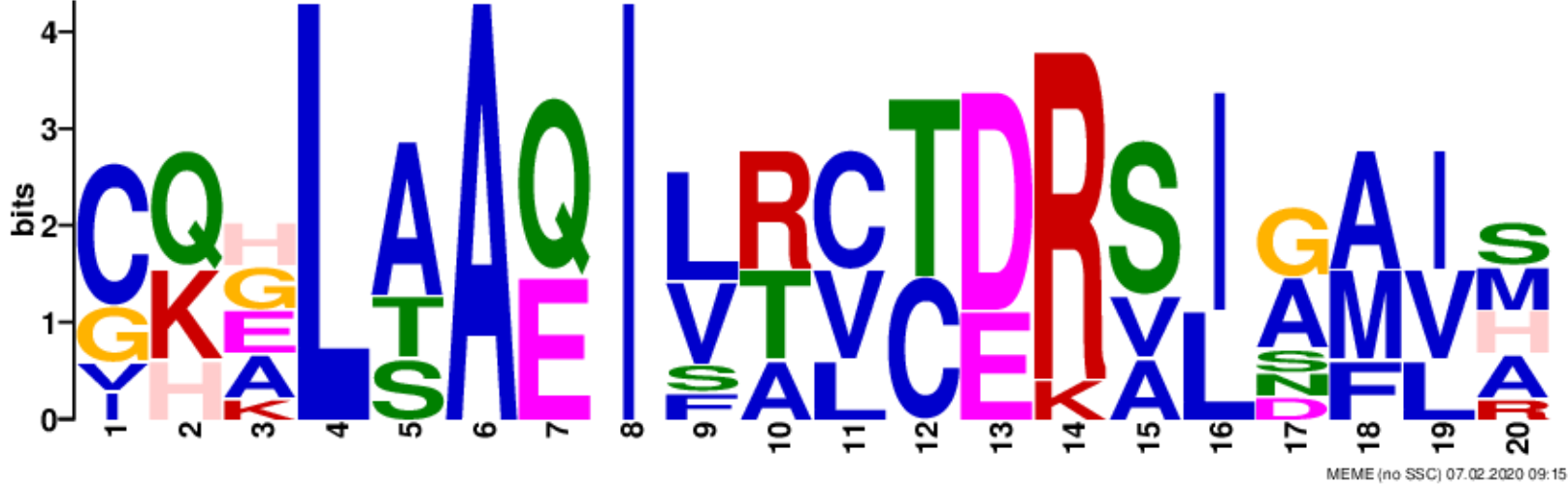

Motif 9

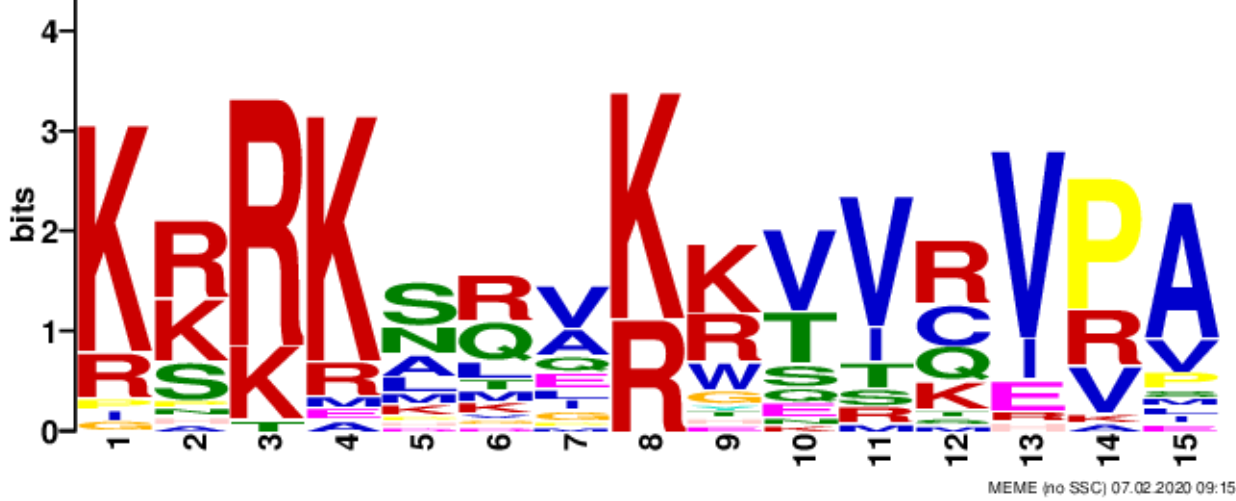

Motif 10

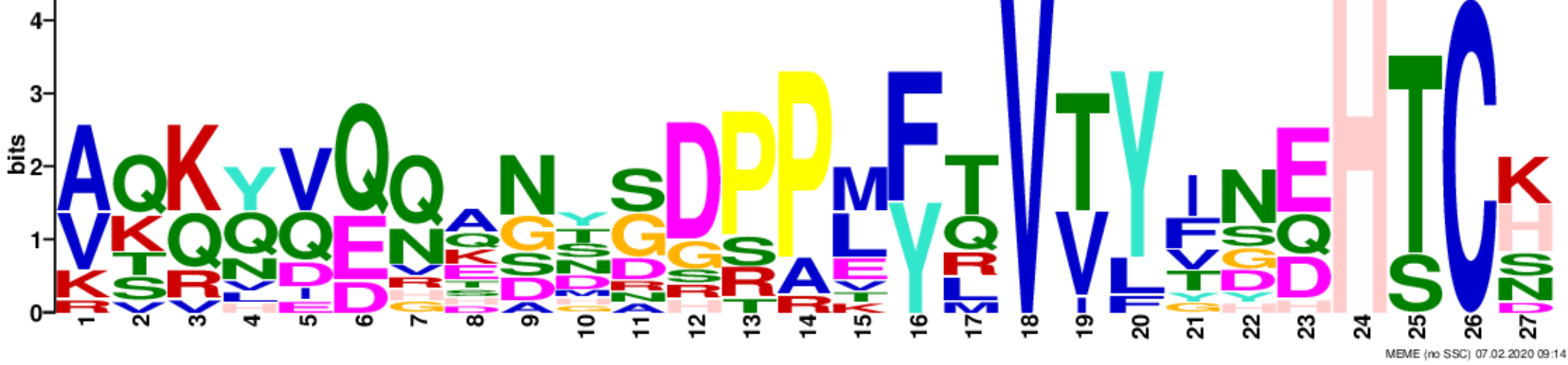

Supplement: Supplemental Information 7 [file peerj-09-11919-s007.pdf]
